# DNA methylation partially mediates the associations of Mediterranean diet scores with MS onset-risk: Results from the Ausimmune case–control study

**DOI:** 10.1177/13524585261462517

**Published:** 2026-07-21

**Authors:** Mehari Woldemariam Merid, Alex Eisner, Daniel J Park, Sam Tanner, Ingrid van der Mei, Vicki E Maltby, Bruce V Taylor, Jeannette Lechner-Scott, Rodney J Scott, Sarah M Thomson, Simon A Broadley, Ellen Morwitch, Trevor J Kilpatrick, Anne-Louise Ponsonby, Steve Simpson-Yap, Ausimmune Investigators Group

**Affiliations:** The Florey Institute of Neuroscience and Mental Health, The University of Melbourne, Parkville, VIC, Australia; Department of Epidemiology and Biostatistics, Institute of Public Health, College of Medicine and Health Sciences, University of Gondar, Gondar, Ethiopia; The Florey Institute of Neuroscience and Mental Health, The University of Melbourne, Parkville, VIC, Australia; Melbourne Bioinformatics, The University of Melbourne, Parkville, VIC, Australia; Department of Biochemistry and Pharmacology, The University of Melbourne, Parkville, VIC, Australia; The Florey Institute of Neuroscience and Mental Health, The University of Melbourne, Parkville, VIC, Australia; Melbourne Bioinformatics, The University of Melbourne, Parkville, VIC, Australia; Murdoch Children’s Research Institute, The University of Melbourne, Parkville, VIC, Australia; MS Research Flagship, Menzies Institute for Medical Research, University of Tasmania, Hobart, TAS, Australia; School of Medicine and Public Health, Newcastle University, Callaghan, NSW, Australia; Hunter Medical Research Institute, Newcastle University, Callaghan, NSW, Australia; Department of Neurology, John Hunter Hospital, New Lambton Heights, NSW, Australia; MS Research Flagship, Menzies Institute for Medical Research, University of Tasmania, Hobart, TAS, Australia; School of Medicine and Public Health, Newcastle University, Callaghan, NSW, Australia; Hunter Medical Research Institute, Newcastle University, Callaghan, NSW, Australia; Department of Neurology, John Hunter Hospital, New Lambton Heights, NSW, Australia; Hunter Medical Research Institute, Newcastle University, Callaghan, NSW, Australia; School of Biomedical Sciences and Pharmacy, Newcastle University, Callaghan, NSW, Australia; The Florey Institute of Neuroscience and Mental Health, The University of Melbourne, Parkville, VIC, Australia; School of Medicine and Dentistry, Griffith University, Southport, QLD, Australia; The Florey Institute of Neuroscience and Mental Health, The University of Melbourne, Parkville, VIC, Australia; The Florey Institute of Neuroscience and Mental Health, The University of Melbourne, Parkville, VIC, Australia; The Florey Institute of Neuroscience and Mental Health, The University of Melbourne, Parkville, VIC, Australia; Murdoch Children’s Research Institute, The University of Melbourne, Parkville, VIC, Australia; The Florey Institute of Neuroscience and Mental Health, The University of Melbourne, Parkville, VIC, Australia; MS Research Flagship, Menzies Institute for Medical Research, University of Tasmania, Hobart, TAS, Australia; Neuroepidemiology Unit, Melbourne School of Population and Global Health, The University of Melbourne, Carlton, VIC, Australia

**Keywords:** Multiple sclerosis, diet, DNA methylation, mediation, Mediterranean

## Abstract

**Background:**

Diet intake has been implicated in multiple sclerosis (MS) risk; however, the mechanisms are unclear.

**Objective:**

Investigated whether DNA methylation (DNAm) mediated the associations of diet and MS onset-risk.

**Methods:**

We utilised data from the Ausimmune case–control study, comparing 201 MS cases and 342 matched controls. Diet scores were estimated from food frequency questionnaires. Genotypes and DNAm were measured using the Illumina Global Screening Array and MethylationEPIC BeadChip, respectively. The 2432 top-ranked MS-associated cytosine-guanine pairs (CpGs) were dimension-reduced using Weighted-Gene Correlation Network Analysis (WGCNA) to five (A1-A5) DNAm module scores. Diet–MS associations were evaluated using multivariable logistic regression, and mediation through DNAm modules by counterfactual mediation analysis. We conducted stratified analyses by human leukocyte antigen (*HLA*) genotypes.

**Results:**

The alternate Mediterranean diet score (aMED) including unprocessed red meat (aMED-Red) (adjusted odds ratio (aOR) = 0.88; 95% confidence interval (CI) = 0.79–0.98) and Healthy diet scores (aOR = 0.86; 95% CI = 0.75–0.98) were associated with lower MS onset-risk. A5 DNAm module significantly mediated the associations of aMED (30.3%; *p* = 0.027) and aMED-Red (22.1%; *p* = 0.037) scores with MS onset-risk. Stratified by *HLA* genotypes, no differences in diet–MS associations or A5 DNAm-module mediation were seen.

**Conclusion:**

Up to one-third of the associations of Mediterranean diet scores with MS onset-risk were explained by DNAm in genes involved in immune function. These findings provide a better understanding of the underlying MS pathogenesis and inform future research.

## Introduction

Multiple sclerosis (MS) is a complex autoimmune condition of the central nervous system (CNS), manifesting in progressive disability and loss of function.^1^ MS is a multi-factorial condition involving an interplay between genetic, environmental and lifestyle risk factors.^2^ Epstein-Barr virus (EBV) infection, low vitamin D levels/low sun exposure and tobacco smoking have been implicated in MS risk,^3^ while genetic factors, particularly the HLA-DRB1*1501 and *HLA-A:02* variants,^4,5^ have been consistently associated. In addition to these factors, however, an increasing research base suggests roles for dietary factors in MS risk.^6–9^

Diet fundamentally impacts on the breadth of human health and has been associated with multiple chronic health conditions,^10^ including MS.^11^ People living with MS often seek information about diet^12^ and are apt to engage with diet modification.^13^ Although the associations of individual foods/nutrients with MS have been reported,^7,8,14,15^ there is greater plausibility for diet to act in concert to affect chronic diseases like MS.^16^ These are typically evaluated using diet intake scores, including those based on index-analysis (a priori defined and externally applicable) and factor analysis (defined by patterns in the data).^17^ In the subsequent sections, we provide an overview of the diet scores and patterns included in our study.

**Mediterranean diet**: The Mediterranean diet, based upon diet styles in southeastern Europe, has strong evidence for general health benefits,^18^ but also may be beneficial in MS.^19^ The original Mediterranean diet score, developed by Trichopoulou and colleagues^20^ comprises nine diet domains, each scored 0/1 and summated to realise a total score ranging 0–9 (Supplementary Table S1). Subsequently, Fung and colleagues developed the alternative Mediterranean diet score (aMED),^21^ and Black and colleagues later developed the alternative Mediterranean-Red diet score (aMED-Red).^22^ The makes a number of modifications, splitting/merging/removing/modifying food groups, including limiting the meat group to only consider red/processed meat intake from the original Mediterranean diet. The aMED was further modified in our setting to generate the aMED-Red score, now positively scoring unprocessed red meat (defined as beef, lamb, pork and veal) consumption above the sample median.^22^ Differences between these Mediterranean scores are shown in Supplementary Table S2. Given that each score reflects slightly different assumptions regarding food group classification and population relevance, consistency of findings across these scores helps capture complementary operationalisations of Mediterranean diet scores with MS onset-risk.

A recent systematic review and meta-analysis showed that a high adherence to the Mediterranean diet was associated with reduced odds of MS compared to least adherence.^23^ A Swedish population-based case–control study also showed an inverse association between adherence to the Mediterranean diet–MS risk.^24^

In the Australian Multi-centre Study of Environment and Immune Function (Ausimmune) Study,^25^ Black and colleagues^22^ found that higher categories of aMED-Red were associated with reduced risk of first clinical diagnosis of CNS demyelination (first clinical demyelination (FCD)), although similar trends for aMED did not reach significance. Mediterranean diet scores’ associations with MS have also been evaluated in other case–control studies, such as the Iranian study by Sedaghat and colleagues where high versus low tertile of Med score was inversely associated with MS case status (adjusted odds ratio (aOR) = 0.23, 95% confidence interval (CI) = 0.06–0.89).^26^ Two studies using the UK Biobank data showed inverse, but non-significant, associations between aMED and MS risk.^27,28^ On the contrary, a large cohort study found no evidence for an association between diet quality (measured using several indices including, the aMED) and MS risk.^29^ Key considerations in these studies include limited statistical power (e.g. *N* = 94 in Shang et al.^27^), a potential for recall bias and the possibility of reverse causality.

**Dietary Inflammatory Index (DII):** It is a measure of dietary inflammatory potential. Developed by Shivappa and colleagues in 2014,^30^ this score weights intake data on up to 45 inflammation-related dietary parameters with inflammation coefficients. Products are summated to realise DII scores, positive scores indicating inflammatory potential and negative anti-inflammatory potential. In Ausimmune, Mannino and colleagues showed that higher DII was associated with 17% higher FCD risk in females (95% CI = 1.04–1.33), but no association was evident in males.^31^
**Ultra-processed Food (UPF):** Defined by the NOVA framework, UPFs are industrial formulations which are heavily processed to result in foods that are energy-dense but poor in nutrients.^32^ A greater share of diet comprising UPFs has been shown to reduce nutritional adequacy. Higher UPF intake has been associated with increased risks of chronic health conditions^33^ and neurodegenerative disorders.^34^ A cross-sectional Italian study showed that higher UPF consumption was associated with approximately three-fold higher odds of moderate-to-high MS severity.^35^ In Ausimmune, Mannino and colleagues showed that higher UPF consumption was associated with higher MS onset-risk.^36^
**Healthy and Unhealthy dietary factor scores**: Rather than using an externally derived model for scoring, factor analysis defines patterns in the data and scores these. By applying statistical methods like principal component analysis, representative and informative patterns (aka components or factors) can be derived, and scores for each pattern can be estimated for each participant.^37^

Although heterogeneities exist, studies showed that a health/plant-based dietary pattern is generally linked to reduced MS risk compared to plant-based diets.^27,38^ In Ausimmune, Black and colleagues^6^ applied principal component analysis to categorised Food Frequency Questionnaire (FFQ) data, deriving two components – here labelled ‘Healthy’ and ‘Unhealthy’. The Healthy diet pattern was characterised by higher consumption of poultry, grilled/tinned fish, eggs, yellow/red/cruciferous vegetables and legumes, whereas the Unhealthy diet pattern was characterised by higher intakes of red/processed meat and full-fat dairy and less whole grains, nuts and fresh fruit. While the Unhealthy diet score was not associated with FCD risk, a higher Healthy diet score was associated with 25% lower FCD risk per 1SD (95% CI = 0.60–0.94).

The mechanisms underlying diet–MS associations are unclear, but changes in body mass index (BMI),^39^ dyslipidaemia,^40^ inflammation,^41^ oxidative stress,^42^ and altered gut microbiome^43^ have been suggested. A novel mechanism for diet’s associations with MS may lie in epigenetics. Epigenetic mechanisms comprise several biological processes that regulate gene expression without altering the DNA sequence.^44^ Of these, one well-studied mechanism is DNA methylation (DNAm), which refers to the covalent addition of methyl groups to cytosine-guanine pairs (CpGs) along the DNA molecule, particularly within or near the promoter regions of genes.

The use of molecular biomarkers including DNAm profile scores has become increasingly common in systems biology and molecular epidemiology studies employing causal mediation analysis.^45,46^ With this, we could focus analyses on epigenetic variation of MS-associated CpGs with the greatest biological relevance to MS pathophysiology while reducing the dimensionality and multiple-testing burden inherent in methylome-wide data.^47,48^ This strategy enhances biologic interpretability and reduces noise in downstream mediation analyses while retaining focus on MS-relevant epigenetic variation.

Differential DNAm has been shown in MS^49,50^ and between MS cases and controls.^51^ DNAm can also mediate other risk factors’ associations with MS: we recently showed that differential DNAm acts as a significant mediator of several canonical environmental and genetic risk factors for MS, including EBV, low sun, lower vitamin D and HLA-DRB1*1501.^52^ However, whether DNAm mediates the diet and MS onset-risk associations has not been explored.

Therefore, using data from the Ausimmune case–control study, we investigated: (1) diet–MS associations in this participant subgroup; (2) mediation of diet–MS associations by DNAm-module scores; and (3) whether these relationships differ by BMI and *HLA-DR15* and *HLA-A:02* genotypes.

## Methods

### Study design and population

This study utilised data from the Ausimmune Study,^25^ a multicentre incident-matched case–control study conducted from 2003 to 2007 across four regions of Australia: Brisbane, QLD (latitude 27°S), Newcastle, NSW (33°S), Geelong/Western districts of Victoria, VIC (37°S) and Tasmania (43°S). As described previously,^25^ eligible FCD cases included (1) classic first demyelinating events (FDEs; defined as a single, first, episode of clinical symptoms suggestive of CNS demyelination); (2) a first clinically recognised event at presentation, but during which clinical examination revealed a prior undiagnosed episode that, on review, was highly suggestive of CNS demyelination; and (3) a first presentation of primary progressive MS (based on neurologic assessment on study entry). Controls were randomly selected from the Australian Electoral Roll and matched to cases by age (±2 years), sex and study region. Ethics approval was obtained from the nine Human Research Ethics Committees (HRECs) of participating institutions, and written informed consent were obtained from all participants.

The Ausimmune study comprised 282 FCD cases and 558 age/sex/region-matched controls. However, analyses here were limited to the 201 cases who were not still demonstrably clinically isolated syndrome (CIS) by 10-year follow-up and their matched 342 controls, and for whom DNAm and diet intake were measured.

We conducted the present research and the presentation thereof as per the STROBE^53^ and AGReMA guidelines,^54^ as shown in Supplementary Files 1 and 2, respectively.

### Diet measures, dietary index and factor score estimation

The Cancer Council Victoria DQESv2 FFQ queried dietary intake for 101 foods and beverages (recorded on a scale ranging ‘never’ to ‘⩾3 times a day’) in the 12 months prior to the date of interview.^55^ Food/beverages were each converted into grammes/day and specified nutritional parameters (e.g. energy content (kcal/day), nutrients) were estimated by a propriety algorithm.

In addition, we generated a dietary misreporting parameter, equal to the ratio of participants’ energy intake to their estimated basal metabolic rate, as described previously.^56^

From these FFQ data, our group has estimated a number of diet scores, the methods for which are next described.

#### Alternative Mediterranean diet: aMED and aMED-Red

The scoring for aMED and aMED-Red was generally in keeping with the methods by Fung and colleagues.^21^ However, whereas for aMED, one point was assigned for intakes (g/d) of unprocessed and processed red meat below the sex-specific median and, for aMED-Red, the meat component of the aMED was modified to positively score unprocessed red-meat consumption (beef, lamb, pork, veal).^22^ Each score ranged 0–9, with a higher score indicating greater Mediterranean diet engagement.

#### DII/energy-adjusted Dietary Inflammatory Index

Available DII algorithm diet/nutritional parameters (*n* = 26 for DII, *n* = 25 for energy-adjusted Dietary Inflammatory Index (eDII)) were processed by staff at the University of South Carolina using a proprietary algorithm to estimate the DII and eDII scores. As described previously,^30,31^ diet/nutritional parameters were first standardised and then multiplied against inflammatory effect coefficients, and these products were then summated to generate DII and eDII scores for each participant.

#### UPF consumption

As described previously,^36^ 28 FFQ items were used for estimating UPF intake. Intakes in grammes/day were converted to energy-adjusted UPF servings/day.

#### Factor analysis–derived diet scores

As described previously,^6^ principal component analysis with orthogonal rotation was applied to 34 a priori food groupings of FFQ items, deriving two major dietary patterns, here labelled as Healthy and Unhealthy. Scores for each pattern were estimated for each participant.

### Genetic and epigenetic measures

Details regarding the methodology for our genetic and epigenetic measures have been described previously.^51,52^ Briefly, whole blood was obtained from each participant, and DNA extracted using QIAamp DNA Blood Mini (Qiagen, the Netherlands). Genotyping was conducted by the Global Screening Array v2.1 (Illumina, San Diego, CA, USA). DNAm was measured using the Illumina Infinium Human Methylation EPIC BeadChip (Illumina) and processed as described previously.^51^

As described previously,^52^ an Epigenome-Wide Association Study (EWAS) was used to identify CpGs associated with FCD risk. The top 2432 FCD-associated CpGs were then clustered and dimension-reduced using Weighted-Gene Correlation Network Analysis (WGCNA)^57^ into five DNAm modules, represented by their first principal components, and conventinally named A1 to A5, where “A” denotes Ausimmune. Pairwise correlations between CpGs were used to generate a signed network, transformed into a topological overlap matrix and clustered hierarchically to identify modules using dynamic tree cutting. The WGCNA identifies biologically relevant co-methylation networks by clustering CpGs based on their correlation structure rather than associations with the exposure or outcome to derive modules.^58^ This two-stage approach, screening CpGs based on their associations with the exposure and/or outcome, followed by mediation analysis by DNAm scores, is widely used in high-dimensional epigenetic studies.^47,59^ For example, a recent cancer epigenome-wide study preselected CpGs associated with both exposure and outcome prior to mediation analysis.^60^ A similar strategy has also been applied in our previous WGCNA-based work to identify epigenetic mediators of environmental risk factors in MS.^52^

Biological characteristics of DNAm modules were assessed using active sub-network enrichment analysis (pathfindR package in R^61^) using the Reactome and Gene Ontology databases.

### Other measures

Demographic, environmental and lifestyle parameters was obtained from participant questionnaires.^25^ BMI was derived from participants’ heights (m) and weights (kg) by the function weight/height^2^ and categorised.^62^ Serum vitamin D was measured by liquid chromatography–mass spectrometry (LC/MS)^63^ and deseasonalised.^64^ Supplementary Figure 1 illustrates the timing of measurement of participants’ characteristics and exposures relative to FDE onset.

### Data analyses

STATA/SE18 (StataCorp, College Station, TX, USA) and R (version 4.4.3) were used for analyses.

Logistic regression was used to assess associations with case status, and linear regression was used to assess associations with DNAm-module scores. All analyses were adjusted for the matching covariates (age, sex, study site), education, smoking status, energy intake and dietary misreporting, as appropriate. Covariates were selected a priori from the literature and evaluated using established guidelines for model selection.^65^ Other covariates assessed in previous studies in Ausimmune,^6,22,31,36^ including BMI, physical activity, glandular fever history and serum vitamin D levels, did not materially affect results and were not included in multivariable models here (data not shown). We tested exposure-outcome logit-linearity by comparing the adjusted linear logistic model with a restricted cubic spline for each exposure using a likelihood-ratio test (RCS-LRT), with Akaike information criterion (AIC)/Bayesian information criterion (BIC) supplementary checks.^66^

Given the potential for type-1 error, we accounted for multiple comparisons using Holm’s step-down procedure (family-wise error rate (FWER))^67^ and the Benjamini–Hochberg false discovery rate (FDR)^68^ within each set of diet analyses.

Additive and multiplicative interaction between diet indices and either HLA-DRB1*1501 risk genotype or BMI was evaluated versus MS onset-risk. Multiplicative interactions were evaluated using exposure-interaction product terms. Additive interactions were evaluated by four-level exposure-interaction composite terms, from which relative excess risk due to interaction (RERI), attributable proportion due to interaction (AP) and synergy index (SI) statistics were estimated.^69^

#### Mediation analyses

**Figure 1.**
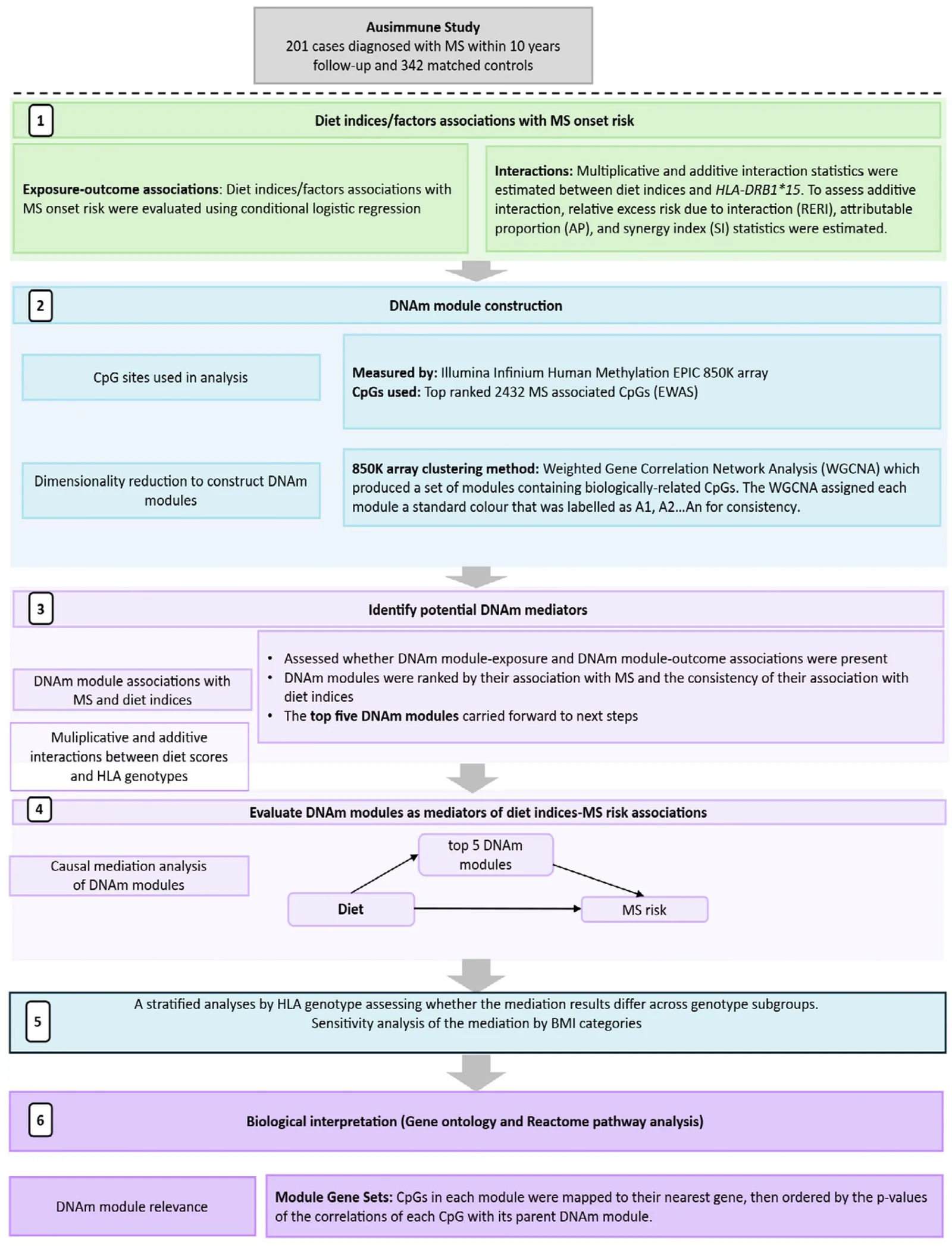
Workflow diagram illustrating the analysis steps adapted from our previous work.^41^ Diet indices/scores were first tested for associations with MS onset-risk and HLA interactions, followed by the construction of DNAm modules from top MS-associated CpGs using WGCNA. The top five DNAm modules were evaluated as potential mediators of diet–MS associations, with stratified and sensitivity analyses by HLA genotype and BMI. Biological interpretation was conducted using gene ontology and Reactome pathway analyses.

As shown in Figure 1, we applied the methods framework from our previous work.^52^ We used counterfactual mediation analysis^70^ (using medflex R package^71^) to evaluate mediation of each diet–MS association, for those with *p*-values < 0.2 in the adjusted model, by each of the five DNAm modules, estimating the total, direct and indirect effects and proportion mediated. When direct and indirect associations were on opposite sides of the null (statistical suppression^72^), proportions mediated were not estimated.

#### Mediation analysis sensitivity analyses

To evaluate the temporal ordering of exposure-mediator-outcome measures, we evaluated several sensitivity analyses suggested previously,^73^ including (1) the associations between FCD-interview durations and exposure/DNAm measures; (2) lag-time restricted analyses (here subgroup analyses limited to classic FDEs) and (3) MS risk-associated gene-set enrichment in DNAm modules. We also evaluated reverse-pathway mediation analyses (MS-mediator-exposure).^74^

We also undertook stratified analyses of DNAm mediation of diet–MS associations by HLA-DRB1*1501 and *HLA-A:02* genotypes; differences were assessed using the Wald intergroup tests.^75^

#### Other sensitivity analyses

We evaluated analyses constrained to those with plausible meal energy contents (males = 800–4000 kcal/day; females = 500–3500 kcal/day).^76^ This subgroup was not materially different in numbers (*n* = 197 cases, 333 controls) nor in associations (data not shown), and thus, we do not discuss these analyses further.

To evaluate the potential effects of unmeasured confounders on our associations, we estimated *E*-values.^77,78^ *E*-values represent the minimum magnitudes that an unmeasured confounder would need to have with both exposure and outcome (for primary associations) or with exposure, outcome and mediator (for mediation analyses), conditional on model covariates to (1) fully explain observed associations or (2) push the inner bound of the 95% CI across the null.

## Results

### Participants’ characteristics

Of the 201 cases and 342 controls included in this analysis, more than three-quarters (78.95%) were female in both cases and controls. The median ages of cases and controls were 38.8 and 39.7 years. Diet quality scores were moderate, including 4–5/9 for Mediterranean scores. Ever smoking (63.5%) and obese BMI (28.0%) were relatively frequent. Genetic risk factors, including HLA-DRB1*1501 (56.72% vs. 28.15%) and *HLA-A:02* (64.56% vs. 49.13%) risk genotypes, were more common among cases than controls. Other participant characteristics are shown in Table 1.

**Table 1.** The socio-demographic, dietary factors and related characteristics of the participants.

Variables	Variable categories	Cases (%/*n*)	Controls (%/*n*)	Difference^a^
Sex	Female	77.61% (156)	78.95% (270)	0.72
	Male	22.39% (45)	21.1% (73)	Ref
Study site	Tasmania	27.86% (56)	21.05% (72)	0.968
	Victoria	24.88% (50)	28.65% (98)	0.432
	Newcastle	9.95% (20)	9.94% (34)	0.394
	Queensland	37.31% (75)	138 (40.35%)	Ref
Ever smoke tobacco	No	36.50% (73)	48.83% (167)	Ref
	Yes	63.50% (127)	51.17% (175)	**0.005**
	Missing	0.97% (2)	0.6% (2)	**0.009**
Education	Year 10 or less	24.8% (51)	31.1% (108)	Ref
	Year 12 or less	49.0% (101)	40.4% (140)	0.24
	University	23.3% (48)	27.1% (94)	0.103
	Missing	2.9% (6)	1.4% (5)	0.099
BMI	Normal	44.3% (89)	38.7% (130)	Ref
	Overweight	27.9% (56)	33.3% (112)	0.19
	Obese	27.9% (56)	28.0% (94)	0.98
HLA-BRB1^a^15	GG	43.28% (87)	71.68% (245)	Ref
	AA/AG^b^	56.72% (114)	28.15% (96)	**<0.001**
HLA-A^a^02	AG	64.56% (133)	49.13% (170)	**<0.001**
	AA^b^	35.44% (73)	50.87% (176)	Ref
Median (interquartile range) for the continuous variables				
Age		38.80 (31.35, 46.55)	39.68 (32.51, 47.62)	0.17
aMED		4 (3, 5)	4 (3, 6)	0.057
aMED-Red		4 (2, 5)	5 (3, 6)	**0.005**
Healthy diet score		−0.57 (-1.16, 0.52)	−0.14 (-1.07, 0.78)	**0.03**
Unhealthy diet score		−0.03 (-1.0, 1.19)	−0.03 (-1.16, 1.07)	0.88
DII		1.83 (0.28, 2.66)	1.36 (0.03, 2.33)	**0.016**
eDII		0.65 (-0.69, 1.76)	0.50 (-0.66, 1.58)	0.38
UPF		5.13 (3.5, 7.27)	5.27 (3.63, 7.87)	0.56

aMED = alternative Mediterranean diet score as per Black et al^22^; aMED-Red = alternative Mediterranean-Red diet score; BMI = body mass index; DII = dietary inflammatory index; eDII = energy-adjusted dietary inflammatory index; UPF = ultra-processed foods. Ref = reference.

a A chi-square/Fisher’s exact test for the differences between the categories in cases and controls.

b Risk variant. Results in boldface denote the statistical significance (*p* < 0.05).

### Associations of diet scores with MS onset-risk

As in Table 2, aMED-Red and Healthy diet scores were each associated with significantly reduced MS onset-risk. Adjusting for educational level and smoking status, all these associations persisted with minimal attenuation and passed the nominal significance threshold. aMED showed a comparable inverse trend, although it did not reach significance. On further adjustment for infectious mononucleosis, serum D25-hydroxyvitamin D (25(OH)D) level, recent sun exposure, physical activity and BMI, the effect estimates were not materially altered (Supplementary Table S3). Assessing effects of potential unmeasured confounders, the elimination *E*-values, overall, were modest (⩽1.6), indicating that only relatively weak unmeasured confounding could explain away the observed diet–MS associations (Supplementary Table S4).

**Table 2.** Associations between dietary scores and MS onset-risk in participants in the Ausimmune study.

Diet scores/factors/UPF consumption	Cases/controls	Model 1		Model 2^a^: further adjusted		FDR-corrected *p*-value
		OR (95% CI)	*p*-value	aOR (95% CI)	*p*-value	
aMED	201/342	0.92 (0.84, 1.02)	0.10	0.93 (0.85, 1.03)	0.14	*0.3150000*
aMED-Red	201/342	**0.88 (0.79, 0.97)**	**0.010**	**0.88 (0.79, 0.98)**	**0.018**	*0.0875000*
UPF	197/333	1.06 (0.96, 1.16)	0.48	1.07 (0.97, 1.18)	0.18	*0.3150000*
DII	197/333	1.11 (0.94, 1.30)	0.24	1.09 (0.92, 1.29)	0.31	*0.3616667*
eDII	197/333	1.08 (0.96, 1.23)	0.19	1.07 (0.94, 1.22)	0.24	*0.3360000*
Healthy diet score	201/342	**0.84 (0.74, 0.96)**	**0.013**	**0.86 (0.75, 0.98)**	**0.025**	*0.0875000*
Unhealthy diet score	201/342	1.01 (0.89, 1.13)	0.91	0.99 (0.88, 1.12)	0.95	*0.9500000*

aMED = alternative Mediterranean diet score; aMED-Red = alternative Mediterranean-Red diet score; DII = Dietary Inflammatory Index; eDII = energy-adjusted Dietary Inflammatory Index; UPF = ultra-processed foods; aOR = adjusted odds ratio; CI = confidence interval. All analyses were conducted using logistic regression, adjusted for meal energy content (kcal/day), dietary misreporting, and matching covariates (age, sex and study site) and estimating aOR (95% CI).

a Adjusted for smoking and educational status, vitamin D and EBNA. No associations met their allocated significant thresholds from both FDR and Holm’s step-down multiple comparisons adjustment. Restricted cubic spline likelihood-ratio test (RCS-LRT) provided no significant non-linearity between diet and FCD case status; information criteria favoured the linear specification.

Results in boldface denote the statistical significance (*p* < 0.05).

### DNAm modules’ associations with diet indices/factors

The aMED and aMED-Red scores were associated with significantly higher A5 DNAm-module score, but no other significant diet score-DNAm associations were observed (Supplementary Table S5). Evaluating the interaction between each of aMED/aMED-Red and A5 DNAm-module and case status, we did not find any significant interactions (data not shown).

### Diet scores and human leukocyte antigen interaction on MS onset-risk

No significant multiplicative interactions were observed between the diet indices and HLA-DRB1*1501 or *HLA-A:02* risk genotypes on MS onset-risk. Evaluating additive interactions, the four-level diet-*HLA* categorical terms consistently showed significant joint effects between worse diet scores and human leukocyte antigen (HLA) risk genotypes on MS onset-risk. However, none of the additive interaction statistics reached statistical significance (Supplementary Table S6).

### Molecular mediation analyses by differential DNAm-module scores

**Figure 2.**
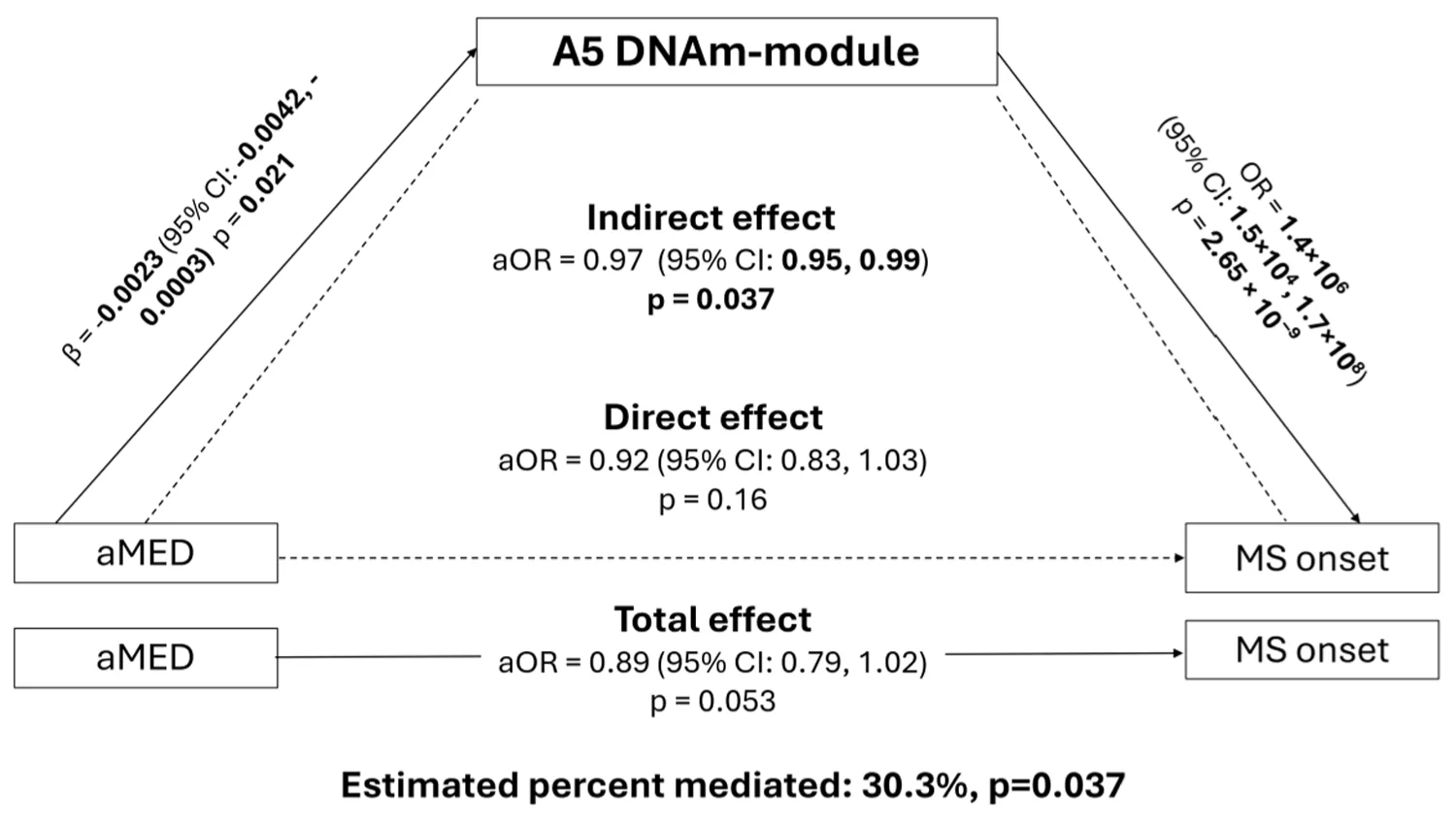
Mediation of the association between aMED score and MS onset-risk by the A5 DNAm module. This figure presents the counterfactual mediation analysis assessing whether differential DNA methylation in the A5 module mediates the protective association between higher alternative Mediterranean diet (aMED) scores and lower MS onset-risk in the Ausimmune study. Shown are total, direct and indirect effects, expressed as odds ratios with 95% confidence intervals. The A5 module accounted for a significant proportion of the association, indicating an epigenetic pathway through which adherence to a Mediterranean-style diet may influence MS onset.

**Figure 3.**
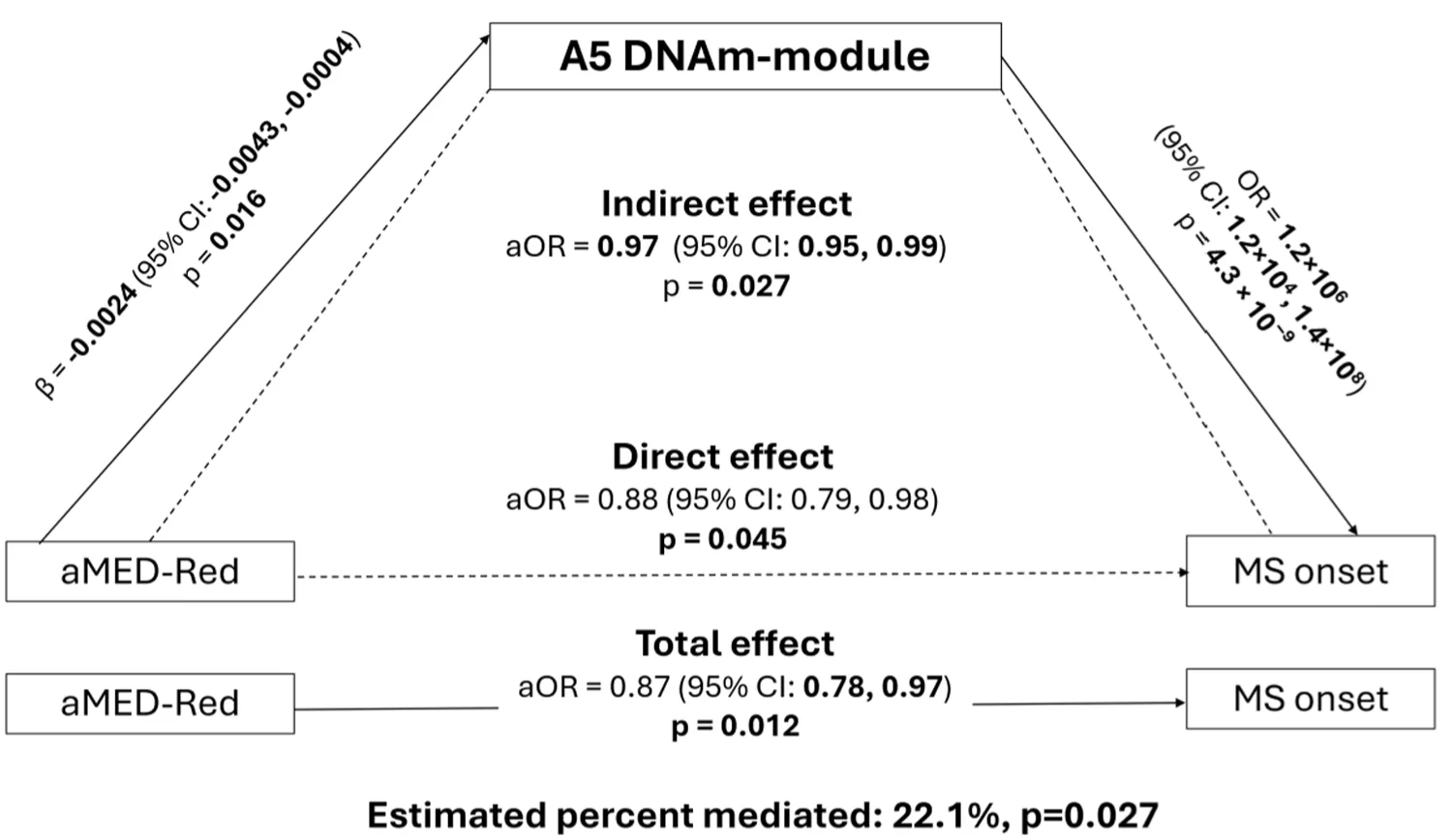
Mediation of the association between aMED-Red score and MS onset-risk by the A5 DNAm module. This figure depicts mediation results evaluating whether the A5 DNA methylation module mediates the inverse relationship between the modified Mediterranean diet score (aMED-Red) and MS onset-risk. Total, direct and mediated indirect effects are presented as odds ratios with 95% confidence intervals. The A5 module significantly mediated part of this association, supporting an epigenetic mechanism linking lower red-meat intake within a Mediterranean dietary pattern to reduced MS onset-risk.

Our mediation analyses showed that the A5 DNAm module score significantly mediated 30.3% of the association of aMED (aOR_indirect_ = 0.97; 95% CI = 0.95–0.99; *p* = 0.027) and 22.1% of the association of aMED-Red (aOR_indirect_ = 0.97; 95% CI = 0.95–0.99; *p* = 0.037) with MS onset-risk (Table 3 and Figures 2 and 3). In addition, the A1-module score mediated 29.3% of the association of the Healthy diet factor score with MS onset-risk, but this did not reach the nominal significance threshold (aOR_indirect_ = 0.95; 95% CI = 0.90–1.00; *p* = 0.052). No significant DNAm mediation of any other diet scores was seen.

**Table 3.** Mediation by the differential DNAm modules of the diet indices and factor scores versus MS onset-risk associations.

Diet scores/indices and UPF consumption	Total effect		Direct effect		Indirect effect		Percent mediated (95% CI)
	aOR (95% CI)	*p*-value	aOR (95% CI)	*p*-value	aOR (95% CI)	*p*-value	
A1							
aMED	0.92 (0.83, 1.01)	0.090	**0.91 (0.83, 0.99)**	**0.034**	1.01 (0.97, 1.05)	0.63	
aMED-Red	**0.88 (0.79, 0.97)**	**0.010**	**0.89 (0.81, 0.97)**	**0.008**	0.99 (0.95, 1.03)	0.64	8.1 (3.9, 29.6)
Healthy diet score	**0.84 (0.74, 0.96)**	**0.009**	**0.88 (0.79, 0.99)**	**0.037**	0.95 (0.90, 1.00)	0.052	29.3 (-5.9, 73.47)
Unhealthy diet score	1.02 (0.91, 1.14)	0.75	1.06 (0.96, 1.17)	0.26	0.96 (0.91, 1.02)	0.16	
A2							
aMED	0.92 (0.83, 1.02)	0.11	0.93 (0.84, 1.02)	0.12	0.98 (0.95, 1.03)	0.60	12.4 (-4.2, 47.8)
aMED-Red	**0.87 (0.79, 0.97)**	**0.009**	**0.89 (0.82, 0.99)**	**0.022**	0.97 (0.93, 1.02)	0.22	19.2 (-8.7, 33.6)
Healthy diet score	**0.84 (0.74, 0.96)**	**0.009**	**0.85 (0.76, 0.96)**	**0.007**	0.99 (0.94, 1.04)	0.70	5.5 (-13.7, 40.5)
Unhealthy diet score	1.02 (0.91, 1.14)	0.76	1.01 (0.90, 1.12)	0.89	1.01 (0.96, 1.06)	0.68	56.5 (-6.9, 78.1)
A3							
aMED	0.92 (0.83, 1.01)	0.079	0.92 (0.84, 1.01)	0.075	0.99 (0.97, 1.03)	0.92	1.9 (-3.8, 34.3)
aMED-Red	**0.88 (0.79, 0.97)**	**0.009**	**0.89 (0.80, 0.97)**	**0.013**	0.99 (0.96, 1.02)	0.53	7.7 (-1.5, 26.8)
Healthy diet score	**0.84 (0.74, 0.96)**	**0.009**	**0.86 (0.76, 0.97)**	**0.017**	0.98 (0.94, 1.02)	0.37	10.7 (-1.7, 44.4)
Unhealthy diet score	1.02 (0.90, 1.14)	0.76	1.02 (0.91, 1.14)	0.74	0.99 (0.96, 1.04)	0.97	
A4							
aMED	0.92 (0.83, 1.01)	0.084	**0.90 (0.82, 0.99)**	**0.036**	1.02 (0.98, 1.04)	0.30	
aMED-Red	**0.88 (0.79, 0.98)**	**0.015**	**0.87 (0.79, 0.96)**	**0.008**	1.01 (0.98, 1.04)	0.58	
Healthy diet score	**0.85 (0.74, 0.95)**	**0.008**	**0.86 (0.7, 06.98)**	**0.020**	0.98 (0.95, 1.01)	0.15	13.9 (-0.7, 43.9)
Healthy diet score	1.02 (0.91, 1.15)	0.72	1.04 (0.93, 1.16)	0.53	0.98 (0.95, 1.02)	0.39	
**A5**							
aMED	0.89 (0.79, 1.02)	0.053	0.92 (0.93, 1.03)	0.16	**0.97 (0.95, 0.99)**	**0.037** ^a^	30.3 (-4.6, 63.5)
aMED-Red	**0.85 (0.76, 0.96)**	**0.006** ^b^	**0.88 (0.79, 0.98)**	**0.045**	**0.97 (0.95, 0.99)**	**0.027** ^a^	22.1 (-0.1, 43.1)
Healthy diet score	**0.84 (0.74, 0.96)**	**0.008** ^b^	**0.85 (0.76, 0.96)**	**0.010**	0.99 (0.96, 1.02)	0.37	8.1 (-1.6, 26.8)
Unhealthy diet score	1.02 (0.90, 1.16)	0.71	1.0 (0.89, 1.13)	0.97	1.02 (0.99, 1.05)	0.18	90.0 (-4.6,137)

aMED = alternative Mediterranean diet score; aMED-Red = alternative Mediterranean-Red diet score; aOR = adjusted odds ratio; CI = confidence interval.

All analyses were conducted using logistic regression, adjusted for meal energy content (kcal/day), dietary misreporting, matching covariates (age, sex and study site), and smoking and educational status, estimating aOR (95% CI). Results in boldface denote the statistical significance (*p* < 0.05). Grey-shaded cells denote when statistical suppression was present (total/direct/indirect associations opposite sides of null).

a Achieved borderline significance after FDR correction (*q*≈0.074).

b Achieved Holm’s step-down multiple comparisons adjustment significance thresholds (*p* = 0.0125 and *p* = 0.016667, respectively).

Stratified by BMI category, there was no consistent dose-dependency in the mediation effects of the A5 DNAm module on diet–MS associations by level of increasing BMI, and no indirect effects reached statistical significance (Supplementary Table S7).

Evaluating the effects of potential unmeasured confounders on mediation analyses, elimination *E*-values for A5 DNAm module’s mediation of aMED and aMED-Red were 1.14 and 1.08, respectively, to render the indirect effects non-significant.

### Mediation sensitivity analyses

To evaluate the temporal ordering of exposure-mediator-outcome measures, we conducted (1) reverse-pathway mediation analyses (case status to mediator to exposure) in which the A5 DNAm module showed no evident mediation, in contrast to the significant effects observed in the primary analyses (exposure to mediator to outcome) mediation analyses (Supplementary Table S8) and (2) the time interval between FCD diagnosis and date of interview did not relate to any of the diet exposures or DNAm scores. In FDE-restricted analyses, the A5 module showed suggestive mediation-consistent in direction with the unrestricted results, although it did not reach nominal significance (data not shown). Gene-set enrichment analyses found no enrichment of MS-associated genes in the A5 DNAm module.

### Mediation analyses, stratified by HLA genotypes

Examining DNAm mediation of diet–MS onset-risk associations in strata of HLA-DRB1*1501 genotype, no differences were found. However, A5 DNAm module mediation of the aMED-Red–MS onset-risk association was only evident among those carrying the protective variant of *HLA-A:02* but not in those without it (45% vs. 7% mediated, *p*_difference_ = 0.11) (Supplementary Table S9).

### Pathway enrichment analyses

The A5 DNAm module comprised 35 CpGs across 35 genes. The Reactome pathways most enriched were ‘GPVI-mediated activation cascade’, ‘PI3K/AKT signalling’, ‘Platelet activation’ and ‘T-Cell Receptor signalling’ (Supplementary Table S10). Gene Ontology pathways most enriched were ‘T-cell activation’, ‘T-cell receptor signalling’, transcription regulation and binding, adaptive immune responses, T-cell differentiation, and B-cell activation and B-cell receptor signalling (Supplementary Table S11). These enrichment analyses consistently highlighted immune-related processes, with strong over-representation of T-cell activation, receptor signalling and *PI3K/AKT* pathways.

## Discussion

Here, we have demonstrated specific mediation of diet–MS associations: only the associations of the aMED and aMED-Red scores were mediated by the same DNAm module. This DNAm module reflected differential DNAm in genes involved in innate and adaptive immune responses and T- and B-cell activation/differentiation. Importantly, these associations were robust to adjustment and did not significantly differ by BMI levels or by HLA-DRB1*1501 or *HLA-A:02* genotypes. These findings indicate plausible functional mechanisms by which Mediterranean diet intake may act upon MS, and that these impacts may be broadly generalisable among people with MS.

The Mediterranean diet – rich in fruits, vegetables, whole grains, fish and healthy fats – provides essential minerals, vitamins and bioactive compounds^79^ and has beneficial effects on multiple health outcomes.^18^ In addition, these dietary components are also known to directly influence gene expression, both directly and via DNAm.^80^ Nutrients enriched in the Mediterranean diet, such as vitamin B, folate, methionine, choline and zinc, are involved in methyl group production and DNAm-related enzymes like DNA methyltransferase.^81^ Emerging evidence underscores the importance of DNAm in the pathogenesis of MS, and there is a high plausibility for DNAm as a mediator underlying the associations of risk factors with MS onset-risk.^82^ The use of DNAm modules constructed using WGCNA prioritises biologically relevant epigenetic variation while reducing the high dimensionality and multiple-testing burden inherent in methylome-wide analyses and improving interpretability.^47,59^ We have recently demonstrated that DNAm modules significantly mediated the associations of several canonical environmental and genetic risk factors with MS onset-risk.^52^ Here, we found that another DNAm module, not previously found to mediate other exposure–MS associations, specifically mediated the associations of Mediterranean diet with MS onset-risk. That only these two Mediterranean diet scores showed similar mediation by the same A5 DNAm-module score indicates that this is not merely a nonspecific effect of diet quality. This A5 DNAm module comprised 35 CpGs in 35 genes involved in innate and adaptive immune function and signalling. While further investigations are required to clarify these relationships, these represent plausible modes by which the Mediterranean diet may affect MS risk via modulation of immune and cellular pathways.

In investigating multiplicative and additive interactions between diet and HLA genotypes and MS onset-risk, including stratified mediation analyses, results were inconsistent and complicate interpretation. The *HLA* genotypes are well-established genetic determinants for MS onset-risk, HLA-DRB1*1501 deleterious and *HLA-A:02* protective.^4,5^ Here, no multiplicative interactions were seen between diet scores and either genotype versus MS risk. However, while diet-HLA + joint associations were significant, none of the additive interaction statistics reached significance. While A5 DNAm-module mediation of aMED-Red-MS association was only seen among those carrying the protective *HLA-A:02* variant, this difference did not reach significance, and no similar difference was seen for the aMED-MS mediation. In addition, no other differences were seen by *HLA-A:02* or by HLA-DRB1*1501. There is plausibility for interactions between diet and HLA genotypes in modulating autoimmune conditions,^83^ but this is the first such study of these dynamics in MS. Replication of these results in other settings is needed to clarify these relationships.

### Strengths, limitations and future directions

This study is the first to investigate the mediating role of DNAm modules in the relationship between diet and MS, providing suggestive evidence relevant to understanding the potential nutri-epigenomic context of MS aetiology. Indeed, the Ausimmune study’s measurement of comprehensive diet intake by FFQ alongside genome-wide DNAm is globally unique, enabling the analyses undertaken here. This study has particular strengths by virtue of its study design: by recruiting cases early in the disease course, and particularly in the early 2000s, when engagement with environmental and lifestyle behaviours was not as common as now, issues of active/passive behaviour change due to diagnosis and disease progression^13^ are reduced compared to contemporary studies.

In addition, the comprehensive measures of diet exposures and covariates in Ausimmune enabled robust assessment of confounding, as well as undertaking stratified analyses by genotype and BMI. However, the *E*-values estimated were of plausible magnitudes, both for diet–MS associations and DNAm mediation of diet–MS associations. However, we evaluated multiple potential confounders, including key known risk factors for MS (EBV, smoking, vitamin D) and those relevant to diet (BMI, physical activity), whose results were robust. While unmeasured confounders beyond these are possible, these analyses give confidence to the likely robustness of these associations.

There are some limitations for this study. First, due to the case–control nature of the design and self-report dietary measures, causal directionality cannot be inferred. To better understand causal directionality, particularly for mediation analyses, for which temporality of exposure-mediator-outcome is fundamental, we undertook a number of supportive statistical analyses, including reverse-pathway mediation analyses, all of which support the diet-DNAm-MS directionality. Nonetheless, we acknowledge the need for substantiation of these associations and mediation thereof in prospective cohort studies.

In addition, a potential limitation of our approach is that the CpGs used to derive DNAm modules were preselected based on their association with MS risk within the same dataset in which mediation analyses were conducted, which may introduce some bias in mediation.^84^ Although this approach is common in studies evaluating dimension-reduced DNAm or other-omic mediators,^60,85,86^ explicit consideration of potential selection bias has been limited. Consequently, the observed mediation effects should be interpreted cautiously and, where feasible, validated in independent datasets or using split-sample approaches. We considered the split-sample approach; however, the available sample size in the Ausimmune dataset was insufficient to support a split while retaining adequate statistical power for mediation analyses.

We also note that due to small sample/subgroup sizes, the effect estimates for the overall mediation results, as well as the stratified analyses of HLA and BMI, are modest and could be unstable. As such, our findings should be interpreted with caution. These constraints are inherent to studies integrating high-dimensional DNAm data and stratification analyses, suggesting a need to evaluate these relationships in larger samples, particularly those with larger recruitment by these factors. In addition, future studies may benefit from alternative statistical approaches with enhanced estimation capabilities.

Finally, our study assessed DNAm in peripheral blood immune cells, rather than in samples from the CNS. While there is functional plausibility for peripheral mechanisms for diet–MS associations,^16^ further exploration of CNS-specific DNAm is needed. In addition, while the identified DNAm modules provide insight into potential epigenetic mechanisms, the lack of transcriptomic or proteomic data limits direct functional validation at the gene or protein level. Integration of DNAm with transcriptomic and proteomic profiling in future studies will help clarify its downstream biological relevance.

## Conclusion

In this study, we found that the Mediterranean diet score, estimated using two measures, was inversely associated with MS onset-risk. Up to one-third of these associations were mediated through common differential DNAm module scores in loci involved in innate and adaptive immune response.

Overall, these findings suggest that lifestyle factors may influence disease risk through plausible epigenetic pathways, informing potential directions for MS research. Diet-related DNAm variations may represent a potential mechanism in MS that warrants further investigation.

## Supplemental Material

sj-doc-1-msj-10.1177_13524585261462517 – Supplemental material for DNA methylation partially mediates the associations of Mediterranean diet scores with MS onset-risk: Results from the Ausimmune case–control study Supplemental material, sj-doc-1-msj-10.1177_13524585261462517 for DNA methylation partially mediates the associations of Mediterranean diet scores with MS onset-risk: Results from the Ausimmune case–control study by Mehari Woldemariam Merid, Alex Eisner, Daniel J Park, Sam Tanner, Ingrid van der Mei, Vicki E Maltby, Bruce V Taylor, Jeannette Lechner-Scott, Rodney J Scott, Sarah M Thomson, Simon A Broadley, Ellen Morwitch, Trevor J Kilpatrick, Anne-Louise Ponsonby and Steve Simpson-Yap in Multiple Sclerosis Journal

sj-docx-1-msj-10.1177_13524585261462517 – Supplemental material for DNA methylation partially mediates the associations of Mediterranean diet scores with MS onset-risk: Results from the Ausimmune case–control study Supplemental material, sj-docx-1-msj-10.1177_13524585261462517 for DNA methylation partially mediates the associations of Mediterranean diet scores with MS onset-risk: Results from the Ausimmune case–control study by Mehari Woldemariam Merid, Alex Eisner, Daniel J Park, Sam Tanner, Ingrid van der Mei, Vicki E Maltby, Bruce V Taylor, Jeannette Lechner-Scott, Rodney J Scott, Sarah M Thomson, Simon A Broadley, Ellen Morwitch, Trevor J Kilpatrick, Anne-Louise Ponsonby and Steve Simpson-Yap in Multiple Sclerosis Journal

sj-docx-2-msj-10.1177_13524585261462517 – Supplemental material for DNA methylation partially mediates the associations of Mediterranean diet scores with MS onset-risk: Results from the Ausimmune case–control study Supplemental material, sj-docx-2-msj-10.1177_13524585261462517 for DNA methylation partially mediates the associations of Mediterranean diet scores with MS onset-risk: Results from the Ausimmune case–control study by Mehari Woldemariam Merid, Alex Eisner, Daniel J Park, Sam Tanner, Ingrid van der Mei, Vicki E Maltby, Bruce V Taylor, Jeannette Lechner-Scott, Rodney J Scott, Sarah M Thomson, Simon A Broadley, Ellen Morwitch, Trevor J Kilpatrick, Anne-Louise Ponsonby and Steve Simpson-Yap in Multiple Sclerosis Journal

sj-png-1-msj-10.1177_13524585261462517 – Supplemental material for DNA methylation partially mediates the associations of Mediterranean diet scores with MS onset-risk: Results from the Ausimmune case–control study Supplemental material, sj-png-1-msj-10.1177_13524585261462517 for DNA methylation partially mediates the associations of Mediterranean diet scores with MS onset-risk: Results from the Ausimmune case–control study by Mehari Woldemariam Merid, Alex Eisner, Daniel J Park, Sam Tanner, Ingrid van der Mei, Vicki E Maltby, Bruce V Taylor, Jeannette Lechner-Scott, Rodney J Scott, Sarah M Thomson, Simon A Broadley, Ellen Morwitch, Trevor J Kilpatrick, Anne-Louise Ponsonby and Steve Simpson-Yap in Multiple Sclerosis Journal
