# Supplementary material for: DNA methylation partially mediates the associations of Mediterranean diet scores with MS onset-risk: Results from the Ausimmune case–control study
Source: Mult Scler. 2026 Jul 21;32(10):1022–37. doi: 10.1177/13524585261462517 (PMC13538921; doi:10.1177/13524585261462517)
Supplement: Supplementary file 2 [file sj-docx-2-msj-10.1177_13524585261462517.docx]

**Title:** DNA methylation partially mediates the associations of Mediterranean diet scores with MS onset-risk: results from the Ausimmune case-control study

**Running head**: Mediterranean diet, methylation, and MS

Authors:

Mehari Woldemariam Merid^1^, Alex Esiner^1^, Daniel J Park^2,3^, Sam Tanner^1,2,4^, Ingrid van der Mei^5^, Vicki E Maltby^6,7,8^, Bruce V Taylor^5^, Jeannette Lechner-Scott^6,7,8^, Rodney J. Scott^7,9^, Sarah M Thomson^1^, Simon A Broadley^10^, Ellen Morwitch^1^, Trevor Kilpatrick^1^, Anne-Louise Ponsonby^1,4^, Ausimmune Investigators Group^11^, Steve Simpson-Yap*^1,5,12^

Affiliations

1. The Florey Institute of Neuroscience and Mental Health, The University of Melbourne, Parkville, Australia;
2. Melbourne Bioinformatics, The University of Melbourne, Parkville, Australia;
3. Department of Biochemistry and Pharmacology, The University of Melbourne, Parkville, Australia;
4. Murdoch Children’s Research Institute, The University of Melbourne, Parkville, Australia;
5. MS Research Flagship, Menzies Institute for Medical Research, University of Tasmania, Hobart, Australia;
6. School of Medicine and Public Health, Newcastle University, Callaghan, Australia;
7. Hunter Medical Research Institute, Newcastle University, Callaghan, Australia;
8. Department of Neurology, John Hunter Hospital, New Lambton Heights, Australia;
9. School of Biomedical Sciences and Pharmacy, Newcastle University, Callaghan, Australia;
10. School of Medicine and Dentistry, Gold Coast Campus, Griffith University, Southport, Australia;
11. The full list of people in the Ausimmune Investigators Group is included in the Acknowledgements;
12. Neuroepidemiology Unit, Melbourne School of Population & Global Health, The University of Melbourne, Carlton, Australia.

*Corresponding author:

Dr Steve Simpson-Yap

The Florey Institute of Neuroscience and Mental Health

30 Royal Parade

The University of Melbourne

Parkville, VIC 3052

Australia

[steve.simpsonyap@florey.edu.au](mailto:steve.simpsonyap@florey.edu.au)

**Supplementary tables**

**Table S1**: Mediterranean Diet Score components and scoring (0-9) as per Trichopoulou A and colleagues(1)

**Table S2**: Components, scoring criteria and scoring cut-off points for the aMED and the aMED-Red indices as per Black and colleagues (2).

**Table S3**: Associations between dietary scores and MS onset-risk with further adjustment for factors in participants in the Ausimmune Study

**Table S4:** E-values with 95% confidence intervals for diet scores and MS onset-risk computed from the odds ratios to evaluate unmeasured confounders.

**Table S5:** Associations of modules A1 and A5 DNAm modules with diet indices/factors

**Table S6A.** Diet-*HLA-DBR1*1501* interaction**; Table S6B.** Diet-*HLA-A:02* interaction

**Table S7:** Sensitivity analysis by BMI categories (normal, overweight, obese) of the mediation result by A5 DNAm module

**Table S8**: Reverse pathway mediation by A5 DNAm module for the association of the diet indices/factor scores vs MS onset-risk

**Table S9**: A stratified analysis by HLA-DRB1*15 and HLA-A:02 genotypes of the association between the diet indices/factors and MS onset risk mediation by A5 DNAm module of the Ausimmune study.

**Table S10**. Gene Ontology (GO) enrichment analysis

**Table S11**. Reactome pathway enrichment analysis

**Each of the supplementary tables is presented as follows.**

**Table S1**. Mediterranean Diet Score components and scoring (0-9) as per Trichopoulou A and colleagues. *Components and scoring of the original Mediterranean Diet Score (MDS) as defined by Trichopoulou et al(1). Beneficial domains receive 1 point when intake is at or above the sex‑specific median; detrimental domains receive 1 point when intake is below the median. The fat quality metric is the monounsaturated‑to‑saturated fat ratio. Moderate ethanol intake (women 5–25 g/day; men 10–50 g/day) receives 1 point.* Total score is the sum of domain points (0–9); higher scores indicate greater adherence to the traditional Mediterranean diet pattern

| **Component (domain)** | **Direction** | **Scoring rule (sex-specific medians)** | **Notes/examples** |
| --- | --- | --- | --- |
| Vegetables | Beneficial | 1 if intake ≥ sex-specific median; else 0 | Potatoes typically excluded in original specs |
| Legumes | Beneficial | 1 if ≥ median; else 0 | Beans, lentils, peas |
| Fruits & nuts | Beneficial | 1 if ≥ median; else 0 | Fresh fruit + nuts |
| Cereals | Beneficial | 1 if ≥ median; else 0 | Whole grains and traditional cereals emphasized |
| Fish & seafood | Beneficial | 1 if ≥ median; else 0 | All fish/seafood |
| Dairy products | Detrimental | 1 if < median; else 0 | Milk, cheese, yogurt (as a summed group) |
| Meat & meat products | Detrimental | 1 if < median; else 0 | Red/processed meats and poultry combined |
| Fat quality (MUFA:SFA ratio) | Beneficial | 1 if ≥ median; else 0 | Proxy for olive‑oil–rich fat pattern |
| Ethanol (alcohol) | Moderate | 1 if women: 5–25 g/day; men: 10–50 g/day; else 0 | Only moderate intake receives 1 point |
| **Abbreviation:** MUFA=mono-unsaturated fatty acid; SFA=saturated fatty acid | | | |

**Table S2**: Components, scoring criteria and scoring cut-off points for the aMED and the aMED-Red indices as per Black and colleagues (2).

| Components | Foods included | Scoring criteria^1^ | Scoring cut-off points^2^ | |
| --- | --- | --- | --- | --- |
|  |  |  | Men | Women |
| Fruit | Oranges, apples, pears, bananas, melons, pineapple, strawberry, apricots, peaches, mango, avocado, tinned fruit, fruit juice | Intake (g/day) > median of controls | 234.8 | 200.1 |
| Vegetables | Tomatoes, peppers, lettuce, cucumber, celery, beetroot, carrots, cabbage, cauliflower, broccoli, spinach, peas, green beans, sprouts, pumpkin, onion, garlic, mushroom, zucchini | Intake (g/day) > median of controls | 114.3 | 128.1 |
| Legumes | Baked beans, tofu, other beans | Intake (g/day) > median of controls | 4.6 | 5.0 |
| Nuts | Nuts and peanut butter | Intake (g/day) > median of controls | 2.6 | 2.5 |
| Wholegrains | High fiber bread, wholegrain bread, rye bread, multigrain bread, All Bran, Bran Flakes, Weetbix, porridge, muesli | Intake (g/day) > median of controls | 60.0 | 79.2 |
| Fish | Grilled, fried and tinned | Intake (g/day) > median of controls | 32.6 | 22.8 |
| Red and processed meat^3^ | Beef, veal, lamb, pork, bacon, ham, salami, sausages, hamburger | Intake (g/day) < median of controls | 129.2 | 83.5 |
| Unprocessed red meat^4^ | Beef, veal, lamb, pork | Intake (g/day) within specified range | 32.5-97.5 | 32.5-97.5 |
| MUFA:SFA | MUFA (g/day): SFA (g/day) | Ratio > median of controls | 0.9 | 0.9 |
| Alcohol | Alcohol (ethanol) from beer, red/white/fortified wine, spirits | Intake (g/day) within specified range | 10-25 | 5-15 |
| **Notes:** ^1^For a score of 1 vs. 0; ^2^Sex-specific median intake for control participants (n=451); specific range (g/day) for unprocessed red meat and alcohol; ^3^aMED only; ^4^aMED-Red only aMED, alternate Mediterranean diet score; aMED-Red, alternative Mediterranean diet score with unprocessed red meat. | | | | |

***Supplementary Table S3****. Associations between dietary scores and MS onset-risk with further adjustment for factors in participants in the Ausimmune Study*

| **Diet scores/factors/UPF consumption** | **Cases/controls** | **Model 1** | | **^a^Model 2** | | **^b^Model 3** | |
| --- | --- | --- | --- | --- | --- | --- | --- |
|  |  | **OR (95% CI)** | **P-value** | **aOR (95% CI)** | **P-value** | **aOR (95% CI)** | **P-value** |
| aMED | 201/342 | 0.92 (0.84, 1.02) | 0.10 | 0.93 (0.85, 1.03) | 0.14 | 0.93 (0.84, 1.03) | 0.18 |
| aMED-Red | 201/342 | **0.88 (0.79, 0.97)** | **0.010** | **0.88 (0.79, 0.98)** | **0.018** | **0.88 (0.79, 0.98)** | **0.019** |
| UPF | 197/333 | 1.06 (0.96, 1.16) | 0.48 | 1.07 (0.97, 1.18) | 0.18 | 1.08 (0.98, 1.19) | 0.13 |
| DII | 197/333 | 1.11 (0.94, 1.30) | 0.24 | 1.09 (0.92, 1.29) | 0.31 | 1.06 (0.89, 1.27) | 0.48 |
| eDII | 197/333 | 1.08 (0.96, 1.23) | 0.19 | 1.07 (0.94, 1.22) | 0.24 | 1.06 (0.93, 1.22) | 0.35 |
| Healthy diet score | 201/342 | **0.84 (0.74, 0.96)** | **0.013** | **0.86 (0.75, 0.98)** | **0.025** | **0.86 (0.79, 0.98)** | **0.041** |
| Unhealthy diet score | 201/342 | 1.01 (0.89, 1.13) | 0.91 | 0.99 (0.88, 1.12) | 0.95 | 0.93 (0.84, 1.103) | 0.94 |
| **Abbreviations**: aMED=alternative Mediterranean diet score; aMED-Red=alternative Mediterranean-Red diet score; DII=Dietary Inflammatory Index; eDII= energy adjusted Dietary Inflammatory Index; UPF=ultra-processed foods; aOR=adjusted odds ratio; CI=confidence interval.  All analyses were conducted using logistic regression, adjusted for meal energy content (kcal/day), dietary misreporting, and matching covariates (age, sex and study site), estimating aOR (95%CI). ^a^adjusted for smoking and educational status. ^b^Further adjusted for infectious mononucleosis, serum 25(OH)D level, recent sun exposure, physical activity, and BMI.  No associations met their allocated significant thresholds from Holm’s step-down multiple comparisons adjustment. Restricted cubic spline likelihood-ratio test (RCS-LRT) provided no significant non-linearity between diet and FCD case status; information criteria favoured the linear specification.  Results in boldface denote statistical significance (p<0.05). | | | | | | | |

**Table S4:** E-values with 95% confidence intervals for diet scores and MS onset-risk computed from the odds ratios to evaluate unmeasured confounders. E-values represent the minimum strength of association that an unmeasured confounder would need to have with both the exposure and the outcome, above and beyond the measured covariates, to explain away the observed association. For protective associations (OR<1), E-values were calculated using the reciprocal of the OR. When the 95% CI includes 1, the CI-based E-value is 1.00. Overall, the E-values were modest (≤1.6), indicating that only relatively weak unmeasured confounding could explain away the observed diet–MS associations.

| **Diet score / factor** | **aOR** | **95% CI (LCL)** | **95% CI (UCL)** | **P-value** | **E-value** | **E-value (CI)** |
| --- | --- | --- | --- | --- | --- | --- |
| aMED | 0.93 | 0.85 | 1.03 | 0.170 | 1.36 | 1.00 |
| aMED-Red | 0.88 | 0.79 | 0.98 | 0.018 | 1.53 | 1.16 |
| UPF | 1.07 | 0.97 | 1.18 | 0.180 | 1.34 | 1.00 |
| DII | 1.09 | 0.92 | 1.29 | 0.310 | 1.40 | 1.00 |
| eDII | 1.07 | 0.94 | 1.22 | 0.240 | 1.34 | 1.00 |
| Healthy diet score | 0.86 | 0.75 | 0.98 | 0.025 | 1.60 | 1.16 |
| Unhealthy diet score | 0.99 | 0.88 | 1.12 | 0.950 | 1.11 | 1.00 |
| **Abbreviations**: aMED=alternative mediterranean diet score as per Black and colleagues 2019; aMED-Red= alternative Mediterranean-Red diet score; DII=dietary inflammatory index; eDII= energy adjusted dietary inflammatory index; UPF=ultra-processed foods | | | | | | |

**Table S5:** Associations of modules A1 and A5 DNAm modules with diet indices/factors

|  | **A5** | | **A1** | |
| --- | --- | --- | --- | --- |
|  | **β (95%CI)** | **p-value** | **β (95%CI)** | **p-value** |
| aMED | -0.002279 (-0.004211, -0.0003479) | **0.021** | 0.0004425 (-0.001473, 0.002358) | 0.65 |
| Amed-Red | -0.002391 (-0.004339, -0.0004439) | **0.016** | -0.0004559 (-0.002388, 0.001476) | 0.64 |
| Healthy (prudent) diet score | 0.0008991 (-0.001002, 0.002801) | 0.35 | -0.0022 (-0.004535, 0.000136) | 0.065 |
| Unhealthy diet score | -0.001073 (-0.003445, 0.001299) | 0.37 | -0.001686 (-0.004009, 0.000636) | 0.15 |
| DII | 0.001681 (-0.0006722, 0.004034) | 0.16 | 0.001401 (-0.00187, 0.004672) | 0.40 |
| eDII | 0.0009365 (-0.001601, 0.003474) | 0.47 | 0.0008737 (-0.001646, 0.003393) | 0.49 |
| UPFs | 0.1621 (-0.002009, 0.004582) | 0.44 | -0.0007493 (-0.002638, 0.001139) | 0.44 |
| **Abbreviations**: aMED=alternative mediterranean diet score as per Black and colleagues 2019; aMED-Red= alternative Mediterranean-Red diet score; DII=dietary inflammatory index; eDII= energy adjusted dietary inflammatory index; UPF=ultra-processed foods; OR=odds ratio; CI=confidence interval. **Notations:** All analyses were conducted using logistic regression (matched on age, sex and study site). Results in boldface denote statistical significance (p<0.05). | | | | |

**Table S6A.** Diet-*HLA-DBR1*1501* interaction: Joint associations of dietary indices and *HLA-DRB115:01 with MS onset risk, with **high diet score and HLA-DRB115:01* negativity** as the reference. Odds ratios with 95% CIs are shown, along with additive (RERI, AP, SI) and multiplicative interaction estimates. Poorer diets and *HLA-DRB1*15:01* positivity were each associated with higher MS risk; interaction estimates were small and generally non-significant (RERI≈0, AP≈0, SI≈1; multiplicative term p>0.05).

| **Diet-HLA-DRB1*1501 interactions** | **Cases/controls (% cases)** | **aOR (95%CI)** | **P-value** |
| --- | --- | --- | --- |
| aMED^high^_,_ HLA-DRB1*1501_negative_ | 34/104(24.64%) | Ref |  |
| aMED_low,_ HLA-DRB1*1501_negative_ | 48/122(28.24%) | 1.24(0.72, 2.14) | 0.45 |
| aMED^high^_,_ HLA-DRB1*1501_positive_ | 51/41(55.43%) | **3.68 (2.02, 6.70)** | **p<0.001** |
| aMED_ow,_ HLA-DRB1*1501 _positive_ | 56/44(56%) | **3.85 (2.08, 7.13)** | **p<0.001** |
| **Additive interaction:** RERI (95%CI): -1.85(-4.40, 0.69), p=0.15; AP (95%CI): -1.59 (-4.86, 1.68), p=0.34; SI (95%CI):0.29(-0.04, 0.62), p=0.083). **Multiplicative interaction: aOR** (95%CI): 1.29 (0.57, 2.95), p=0.54 | | | |
| aMED-Red^high^_,_ HLA-DRB1*1501_negative_ | 32/115(21.77%) | Ref |  |
| aMED-Red_low,_ HLA-DRB1*1501_negative_ | 50/111 (31.06%) | 1.60 (0.91, 2.85) | 0.11 |
| aMED-Red^high^, HLA-DRB1*1501_positive_ | 46/42(52.27%) | **3.94(2.11, 7.34)** | **p<0.001** |
| aMED-Red_low,_ HLA-DRB1*1501 _positive_ | 61/43(58.65%) | **4.55(2.51, 8.28)** | **p<0.001** |
| **Additive interaction:** RERI (95%CI): 0.42(-1.91, 2.72), p=0.73; AP (95%CI): 0.24 (-0.21, 0.68), p=0.29; SI (95%CI):**1.41 (0.36, 2.46, p=0.008). Multiplicative interaction: aOR** (95%CI): 1.39 (0.59, 3.25), p=0.75 | | | |
| Healthy diet^high^_,_ HLA-DRB1*1501_negative_ | 35/122(22.29%) | Ref |  |
| Healthy diet_low,_ HLA-DRB1*1501_negaitive_ | 47/104(31.13%) | 1.72(0.96, 3.08) | 0.066 |
| Healthy diet^high^_,_ HLA-DRB1*1501_positive_ | 48/45(51.61%) | **3.45(1.92, 6.19)** | **p<0.001** |
| Healthy diet_low,_ HLA-DRB1*1501 _positive_ | 59/40(59.60%) | **5.49(2.95, 10.20)** | **p<0.001** |
| **Additive interaction** RERI (95%CI): 1.31(-1.58, 4.20), p=0.89; AP (95%CI): 0.11 (-0.49, 0.72), p=0.72; SI (95%CI): 1.41 (-0.08, 3.0, p=0.063). **Multiplicative interaction: aOR** (95%CI): 1.08 (0.49, 2.42), p=0.84 | | | |
| Unhealthy diet_low,_ HLA-DRB1*1501_negative_ | 45/110(29.03%) | Ref |  |
| Unhealthy diet^high^_,_ HLA-DRB1*1501_negative_ | 37/116(24.18%) | 0.76(0.44, 1.31) | 0.32 |
| Unhealthy diet_low,_ HLA-DRB1*1501_positive_ | 51/45(53.12%) | **2.74(1.58, 4.75)** | **p<0.001** |
| Unhealthy diet^high^_,_ HLA-DRB1*1501 _positive_ | 56/40(58.33%) | **3.19(1.82,5.58)** | **p<0.001** |
| **Additive interaction:** RERI (95%CI): 0.69(-1.12, 2.51), p=0.75; AP (95%CI): 0.22 (-0.30, 0.73), p=0.41; SI (95%CI):**1.46 (0.18, 2.35, p=0.046). Multiplicative interaction: aOR** (95%CI): 1.53(0.69, 3.39), p=0.29 | | | |
| dii_low,_ HLA-DRB1*1501_negative_ | 38/116(24.68%) | **Ref** |  |
| Dii^high^_,_ HLA-DRB1*1501_negative_ | 42/101(29.37%) | 1.18(0.62,2.25) | p=0.62 |
| Dii_low,_ HLA-DRB1*1501_positive_ | 42/41(50.60%) | **3.03(1.66,5.56)** | **p<0.001** |
| dii^high^_,_ HLA-DRB1*1501 _positive_ | 62/39(61.39%) | **4.39 (2.27,8.50)** | **p<0.001** |
| **Additive interaction:** RERI (95%CI): 1.18(-1.49, 3.84), p=0.39; AP (95%CI): 0.27 (-0.24, 0.77), p=0.7;9 SI (95%CI):**1.53 (0.06, 2.99, p=0.041). Multiplicative interaction: aOR** (95%CI): 1.23 (0.51, 2.93), p=0.64 | | | |
| edii_low,_ HLA-DRB1*1501_negative_ | 38/109(25.85%) | Ref |  |
| edii^high^_,_ HLA-DRB1*1501_negaitive_ | 42/108(28.0%) | 1.30 (0.74, 2.29) | p=0.3 |
| edii_low,_ HLA-DRB1*1501_positive_ | 48/44(52.17%) | **3.18(1.75, 5.79)** | **p<0.001** |
| edii^high^_,_ HLA-DRB1*1501 _positive_ | 56/36(60.87%) | **4.95(2.61, 9.38)** | **p<0.001** |
| **Additive interaction:** RERI (95%CI): 0.42(-1.91, 2.72), p=0.73; AP (95%CI): 0.23 (-0.15, 0.75), p=0.19; SI (95%CI):**1.58 (0.23, 2.95, p=0.022). Multiplicative interaction: aOR** (95%CI): 1.19 (0.52, 2.74), p=0.68 | | | |
| UPFs_low,_ HLA-DRB1*1501_negative_ | 46/112(29.11%) | Ref |  |
| UPFs^high^_,_ HLA-DRB1*1501_negative_ | 34/105 (24.46%) | 1.0(0.52,1.92) | 0.99 |
| UPFs_low,_ HLA-DRB1*1501_positive_ | 51/38 (57.30%) | **3.20(1.73, 5.89)** | **p<0.001** |
| UPFs^high^_,_ HLA-DRB1*1501 _positive_ | 53/42 (55.79%) | **3.60(1.88, 6.85)** | **p<0.001** |
| **Additive interaction:** RERI (95%CI): 1.41(-1.31, 4.23), p=0.30; AP (95%CI): 0.11 (-0.49, 0.72), p=0.72; SI (95%CI):**1.18 (0.18, 2.35, p=0.046). Multiplicative interaction: aOR** (95%CI): 1.13 (0.47, 2.71), p=0.79 | | | |
| **Abbreviations**: aMED=alternative mediterranean diet score as per Black and colleagues 2019 J; aMED-Red= alternative Mediterranean-Red diet score; DII=dietary inflammatory index; eDII= energy adjusted dietary inflammatory index; pc1=healthy (prudent) diet score; pc2 = unhealthy diet score; UPF=ultra-processed foods; OR=odds ratio; CI=confidence interval; SI= synergy index; RERI=excess risk due to interaction; AP=attributable proportion due to interaction. | | | |

**Table S6B.** Diet-*HLA-A:02* interaction**:** Joint associations of dietary indices and *HLA-A02* with MS onset risk, with high diet score and *HLA-A02* positivity as the reference. Odds ratios with 95% CIs are shown, along with additive (RERI, AP, SI) and multiplicative interaction estimates. Poorer diets and absence of *HLA-A*02* were each linked to higher MS risk, but estimates of interaction were close to null (RERI≈0, AP≈0, SI≈1, non-significant multiplicative terms), indicating that diet and *HLA-A02* contribute largely independent rather than synergistic effect.

| **Diet-** **HLA-A02 interactions** | **Cases/controls (% cases)** | **aOR (95%CI)** | **P_trend_** |
| --- | --- | --- | --- |
| aMed^high^_,_ HLA-A02_positive_ | 39/112 (25.83%) | Ref | **0.0003** |
| aMed_low,_ HLA-A02 _positive_ | 27/46 (36.99%) | 1.59 (0.88, 2.86) |  |
| aMed^high^_,_ HLA-A02 _negative_ | 71/93 (43.29%) | **2.01 (1.36, 3.56)** |  |
| aMed_low,_ HLA-A02_negative_ | 52/60(46.43%) | **2.53 (1.50, 4.27)** |  |
| **Additive interaction:** RERI (95%CI): -0.26(-1.72, 1.19), p=0.72; AP (95%CI): -0.10 (-0.69, 0.73, p=0.15; SI (95%CI): 0.85(0.36, 2.01), p=0.72). **Multiplicative interaction: aOR** (95%CI): 0.72 (0.36, 1.55), p=0.41 | | | |
| aMedRED^high^_,_ HLA-A02_positive_ | 40/121 (24.84%) | Ref | **0.001** |
| aMedRED_low,_ HLA-A02_positive_ | 30/53 (36.14%) | 1.66 (0.93, 2.98) |  |
| aMedRED^high^_,_ HLA-A02_negative_ | 74/102 (42.05%) | **2.41(1.37, 4.23)** |  |
| aMedRED_low,_ HLA-A02_negaitive_ | 54/64(45.76%) | **2.70(1.58, 4.62)** |  |
| **Additive interaction:** RERI (95%CI): -0.37(-1.87, 1.12), p=0.62; AP (95%CI): -0.14 (-0.69, 0.42), p=0.63; SI (95%CI): 0.82 (0.39, 1.72, p=0.59). **Multiplicative interaction: aOR** (95%CI): 0.67(0.32, 1.42), p=0.30 | | | |
| Healthy diet^high^_,_ HLA-A02_positive_ | 30/98(23.44%) | Ref |  |
| Healthy diet_low,_ HLA-A02_posiitive_ | 40/76(34.48%) | **1.89 (1.04, 3.48)** |  |
| Healthy diet^high^_,_ HLA-A02_negative_ | 57/84(40.43%) | **2.34 (1.36, 4.03)** | **<0.001** |
| Healthy diet_low,_ HLA-A02_negative_ | 71/82(46.41%) | **3.21 (1.80, 5.72)** |  |
| **Additive interaction:** RERI (95%CI): -0.03 (-1.64, 1.59), p=0.97; AP (95%CI): -0.01 (-0.51, 0.49), p=0.97; SI (95%CI): 0.98 (0.48, 2.04, p=97). **Multiplicative interaction: aOR** (95%CI): 0.72 (0.34, 1.53), p=0.39 | | | |
| Unhealthy diet_low,_ HLA-A02_positive_ | 38/98(27.94%) | Ref |  |
| Unhealthy diet_low,_ HLA-A02_negative_ | 62/72(46.27%) | **2.23(1.33, 3.74)** |  |
| Unhealthy diet^high^_,_ HLA-A02_positive_ | 32/76(29.63%) | 1.03(0.57, 1.85) |  |
| Unhealthy diet^high^_,_ HLA-A02_negaitive_ | 66/94(41.25%) | **1.81(1.07, 3.05**) | 0.24 |
| **Additive interaction:** RERI (95%CI): -0.45(-1.65, 0.76), p=0.36; AP (95%CI): -0.25 (-0.93, 0.43), p=0.47; SI (95%CI): 0.64 (0.23, 1.82, p=0.41). **Multiplicative interaction: aOR** (95%CI): 1.27(0.60, 2.66), p=0.53 | | | |
| dii_low,_ HLA-A02_positive_ | 25/91 (21.55%) | Ref |  |
| dii_low,_ HLA-A02_negative_ | 60/80 (42.86%) | **2.66(1.51,5.69)** |  |
| dii^high^_,_ HLA-A02_positive_ | 44/74(37.29%) | 1.92(0.99,3.68) |  |
| dii^high^_,_ HLA-A02_negative_ | 65/80 (44.83%) | **2.75 (1.50,5.03)** | **0.008** |
| **Additive interaction:** RERI (95%CI): -0.83(-2.55, 0.89), p=0.35; AP (95%CI): -0.30 (-0.95, 0.35), p=0.39;9 SI (95%CI): 0.68 (0.33, 1.41, p=0.29). **Multiplicative interaction: aOR** (95%CI): 1.85 (0.86, 3.98), p=0.11 | | | |
| UPFs_low,_ HLA-A02_positive_ | 42/88(32.31%) | Ref |  |
| UPFs_low,_ HLA-A02_negative_ | 58/75 (43.6%) | 1.63 (0.97, 2.74) | 0.16 |
| UPFs^high^_,_ HLA-A02_positive_ | 27/77 (25.96%) | 0.88 (0.44, 1.73) |  |
| UPFs^high^_,_ HLA-A02 _negative_ | 67/85 (44.08%) | **2.05 (1.10, 3.81)** |  |
| **Additive interaction:** RERI (95%CI): 0.53(-0.54, 1.61), p=0.33; AP (95%CI): 0.26 (-0.21, 0.75), p=0.28; SI (95%CI): 2.05 (0.31, 13.43), p=0.46). **Multiplicative interaction: aOR** (95%CI): 0.70 (0.33, 1.50), p=0.36 | | | |
| **Abbreviations**: aMed=alternative mediterranean diet score as per Black and colleagues 2019 J; aMed-RED= alternative Mediterranean-Red diet score; DII=dietary inflammatory index; eDII= energy adjusted dietary inflammatory index; UPF=ultra-processed foods; OR=odds ratio; CI=confidence interval; SI= synergy index; RERI=excess risk due to interaction; AP=attributable proportion due to interaction. | | | |

**Table S7:** Sensitivity analysis by BMI categories (normal, overweight, obese) of the mediation result by A5 DNAm module. Sensitivity analysis of diet–MS associations partitioned into total, direct, and indirect (A5-mediated) effects across BMI strata. Odds ratios (95% CIs) and p-values are shown, with percent mediated reported for context. Across strata, indirect effects were non-significant, indicating that A5 DNAm not mediated diet-MS associations differentially by BMI; Patterns in total/direct effects were directionally consistent with the main analyses (e.g., healthier diet signals in normal BMI), but no consistent BMI-specific mediation emerged.

|  | **Total effect** | | **Direct effect** | | **Indirect effect** | | **Percent mediated** |
| --- | --- | --- | --- | --- | --- | --- | --- |
| **BMI categories** | **OR (95%CI)** | **P-value** | **OR (95%CI)** | **P-value** | **OR (95%CI)** | **P-value** |  |
| **Normal** |  |  |  |  |  |  |  |
| aMED | 0.97 (0.94, 1) | 0.06 | 0.97 (0.95, 1.01) | 0.12 | 0.99 (0.98, 1.0) | 0.20 | 16.7 |
| aMED-Red | 0.96 (0.93, 0.99) | **0.016** | 0.96 (0.93, 1.0) | **0.044** | 0.99 (0.98, 1.0) | 0.15 | 12.8 |
| Healthy diet score | 0.84 (0.74, 0.95) | **0.008** | 0.85 (0.75, 0.96) | **0.010** | 0.99 (0.95, 1.02) | 0.37 | 8.6 |
| Unhealthy diet score | 1.02 (0.90, 1.15) | 0.71 | 1.02 (0.88, 1.13) | 0.96 | 1.02 (0.99, 1.05) | 0.18 | 12.6 |
| **Overweight** | | | | | | | |
| aMED | 0.97 (0.97, 1.02) | 0.26 | 0.98 (0.93, 1.02) | 0.46 | 0.99 (0.98, 1.0) | 0.09 | 33.3 |
| aMED-Red | 0.97 (0.92, 1.01) | 0.21 | 0.98 (0.93, 1.02) | 0.37 | 0.99(0.98, 1.0) | 0.062 | 29.5 |
| Healthy diet score | 1.06 (0.96, 1.16) | 0.19 | 1.01 (0.98, 1.03) | 0.37 | 1.08 (0.97, 1.18) | 0.13 | 15.4 |
| Unhealthy diet score | 0.85 (0.75, 0.96) | **0.013** | 0.99 (0.95, 1.01) | 0.34 | 0.84 (0.73, 0.95) | 0.62 | 8.1 |
| **Obese** |  |  |  |  |  |  |  |
| aMED | 1.0 (0.95, 1.0) | 0.89 | 1.0 (0.96, 1.04) | 0.59 | 0.99 (0.97, 1.0) | 0.22 |  |
| aMED-Red | 0.99 (0.94, 1.03) | 0.66 | 0.99 (0.95, 1.03) | 0.97 | 0.99(0.97, 1.01) | 0.27 | 8.4 |
| Healthy diet score | 1.07 (0.96, 1.19) | 0.19 | 1.07 (0.96, 1.17) | 0.18 | 1.00 (0.96, 1.04) | 0.88 | 4.0 |
| Unhealthy diet score | 0.84 (0.74, 0.95) | **0.007** | 0.85 (0.75, 0.95) | **0.006** | 0.99 (0.94, 1.04) | 0.71 | 5.5 |
| **Abbreviations**: aMED=alternative Mediterranean diet score; aMED-Red=alternative Mediterranean-Red diet score; aOR=adjusted odds ratio; CI=confidence interval.  All analyses conducted using logistic regression, adjusted for meal energy content (kcal/day), dietary misreporting, matching covariates (age, sex and study site), and smoking and educational status, estimating aOR (95%CI). Results in boldface denote statistical significance (p<0.05). Gray shaded cells denote when statistical suppression was present (total/direct/indirect associations opposite sides of null). | | | | | | | |

**Table S8:** Reverse pathway mediation by A5 DNAm module for the association of the diet indices/factor scores vs MS onset-risk

|  | **Total effect** | | **Direct effect** | | **Indirect effect** | |  |
| --- | --- | --- | --- | --- | --- | --- | --- |
| **Exposure** | **aOR (95%CI)** | **P-value** | **aOR (95%CI)** | **P-value** | **aOR (95%CI)** | **P-value** | **Prop. Mediated** |
| aMED | 0.81 (0.53, 1.21) | 0.16 | 0.96 (0.93, 0.99) | 0.055 | 0.89 (0.79, 1.02) | 0.053 | 0.29 |
| aMED-Red | 0.88 (0.79, 0.98) | **0.027** | **0.96 (0.93, 0.99)** | **0.024** | 0.85 (0.76, 0.95) | 0.006 | 0.21 |
| Healthy (prudent) diet score | 0.87 (0.76, 1.00) | 0.057 | 0.99 (0.96, 1.02) | 0.64 | 0.86 (0.75, 1.00) | 0.056 | 0.05 |
| Unhealthy diet score | 1.04 (0.90, 1.19) | 0.56 | 1.03 (0.99, 1.07) | 0.062 | 1.07 (0.93, 1.23) | 0.29 | 0.45 |
| **Abbreviations:** aMED=alternative mediterranean diet score as per Black and colleagues 2019 J; aMED-Red= alternative Mediterranean-Red diet score; OR=odds ratio; CI=confidence interval. All analyses adjusted for age, sex, study site, educational level, smoking status, and energy intake. Results in boldface denote statistical significance (p<0.05). | | | | | | | |

**Table S9:** A stratified analysis by HLA-DRB1*15 and HLA-A:02 genotypes of the association between the diet indices/factors and MS onset risk mediation by A5 DNAm module of the Ausimmune study.

|  | ***HLA-DRB1*15* positive** | | | | | | | ***HLA-DRB1*15* negative** | | | | | | | **Difference*** | |
| --- | --- | --- | --- | --- | --- | --- | --- | --- | --- | --- | --- | --- | --- | --- | --- | --- |
|  | **Total effect** | | **Direct effect** | | **Indirect effect** | | | **Total effect** | | **Direct effect** | | **Indirect effect** | | | **p-value** | |
| **Diet indices/factors** | aOR (95%CI) | P-value | aOR (95%CI) | P-value | aOR (95%CI) | P-value | % mediated | aOR (95%CI) | P-value | aOR (95%CI) | P-value | aOR (95%CI) | P-value | % mediated |  |  |
| aMED | 0.95(0.97, 1.02) | 0.15 | 0.99(0.95, 1.02) | 0.34 | 0.99(0.98, 1.0) | 0.14 | 30% | 0.93 (0.81, 1.07) | 0.31 | 0.95 (0.83, 1.1) | 0.51 | 0.97 (0.94, 1.01) | 0.18 | 38% | 0.95 |  |
| aMED-Red | **0.85 (0.73, 0.98)** | **0.021** | 0.87 (0.75, 1.0) | 0.053 | 0.97 (0.93, 1.02) | 0.24 | 18% | 0.91 (0.79, 1.06) | 0.23 | 0.94 (0.82, 1.09) | 0.42 | 0.97 (0.93, 1.01) | 0.11 | 33% | 0.86 |  |
| health diet score | 0.91 (0.78, 1.07) | 0.26 | 0.93 (0.8, 1.08) | 0.35 | 0.98 (0.95, 1.02) | 0.44 | 22% | 0.86 (0.71, 1.05) | 0.14 | 0.88 (0.72, 1.06) | 0.17 | 0.99 (0.95, 1.03) | 0.54 | 7% | 0.66 |  |
| Unhealthy diet score | 0.98 (0.8, 1.2) | 0.85 | 0.95 (0.77, 1.17) | 0.61 | 1.03 (0.97, 1.1) | 0.31 | -1.5% | 0.99 (0.84, 1.17) | 0.91 | 0.98 (0.84, 1.14) | 0.79 | 1.01 (0.97, 1.06) | 0.57 | -1% | 0.53 |  |
|  | ***HLA-A:02* positive** | | | | | | | ***HLA-A:02* negative** | | | | | | | **p-value** | |
| aMED | 0.94 (0.83, 1.06) | 0.31 | 0.98 (0.87, 1.1) | 0.7 | 0.96 (0.92, 1) | 0.063 | 67% | 0.91 (0.77, 1.09) | 0.31 | 0.93 (0.78, 1.11) | 0.44 | 0.98 (0.94, 1.02) | 0.30 | 22% | 0.36 |  |
| aMED-Red | 0.89 (0.79, 1.01) | 0.084 | 0.94 (0.84, 1.06) | 0.34 | **0.95 (0.91, 0.99)** | **0.019** | 45% | 0.87 (0.73, 1.03) | 0.11 | 0.87 (0.73, 1.04) | 0.14 | 0.99 (0.95, 1.03) | 0.57 | 7% | 0.11 |  |
| Healthy diet score | 0.96 (0.81, 1.15) | 0.67 | 0.97 (0.82, 1.15) | 0.73 | 0.99 (0.93, 1.05) | 0.78 | 25% | **0.83 (0.69, 0.99)** | **0.03** | 0.84 (0.7, 1.01) | 0.067 | 0.98 (0.95, 1.01) | 0.24 | 11% | 0.93 |  |
| Unhealthy diet score | 1.02 (0.88, 1.18) | 0.8 | 0.98 (0.85, 1.14) | 0.8 | 1.04 (0.99, 1.09) | 0.13 | 2% | 0.95 (0.78, 1.16) | 0.64 | 0.96 (0.79, 1.17) | 0.68 | 0.99 (0.95, 1.04) | 0.79 | 20% | 0.22 |  |
| **Abbreviations:** aMED= alternative Mediterranean diet; aMED-Red = alternative Mediterranean-red diet. All analyses conducted using logistic regression, adjusted for meal energy content (kcal/day), dietary misreporting, matching covariates (age, sex and study site), and smoking and educational status, estimating aOR (95%CI). Results in boldface denote statistical significance (p<0.05). *Wald test for comparing the HLA positive and negative models. | | | | | | | | | | | | | | | | |

***Table S10****.* Gene Ontology (GO) enrichment analysis of the gene set. Columns show the GO identifier and description (Term_Description), Fold_Enrichment (over-representation relative to background), Support (proportion of mapped genes), lowest_p and highest_p (significance values), and the specific up- and down-regulated genes contributing to each enriched GO category. GO enrichment analysis highlighted strong over-representation of immune-related biological processes, particularly T cell activation, T cell receptor signaling, and adaptive immune response pathways. Core signaling molecules including LCK, LAT, ZAP70, and PIK3CD were recurrent drivers across these categories. In addition, enrichment was observed in leukocyte migration, phosphorylation and kinase activity terms, indicating convergence on intracellular signaling cascades. Transcriptional regulation processes, such as DNA-binding transcription factor activity and chromatin binding, were also significantly enriched, largely driven by SPI1, TAL1, and TCF7L2. Together, these results suggest that both immune effector functions and transcriptional control mechanisms are central to the biology of the identified gene set.

| **ID** | **Term_Description** | **Fold_Enrichment** | **support** | **lowest_p** | **highest_p** | **Up_regulated** | **Down_regulated** |
| --- | --- | --- | --- | --- | --- | --- | --- |
| GO:0042110 | T cell activation | 68.16633 | 0.037037 | 5.02E-06 | 5.02E-06 | PIK3CD, ZAP70, LAT | |
| GO:0061629 | RNA polymerase II-specific DNA-binding transcription factor binding | 12.83652 | 0.027778 | 5.53E-06 | 5.53E-06 | SPI1, TAL1 | TCF7L2 |
| GO:0003700 | DNA-binding transcription factor activity | 6.746838 | 0.009259 | 3.69E-05 | 3.69E-05 | SPI1, TAL1 | TCF7L2 |
| GO:0045121 | membrane raft | 15.56554 | 0.009259 | 3.77E-05 | 0.000812 | LCK, ZAP70, LAT | |
| GO:0004715 | non-membrane spanning protein tyrosine kinase activity | 47.06723 | 0.009259 | 4.49E-05 | 0.035245 | LCK, ZAP70 | |
| GO:0018108 | peptidyl-tyrosine phosphorylation | 26.35765 | 0.009259 | 0.000179 | 0.002195 | LCK, ZAP70 | |
| GO:0004713 | protein tyrosine kinase activity | 21.96471 | 0.009259 | 0.000187 | 0.002548 | LCK, ZAP70 | |
| GO:0050852 | T cell receptor signaling pathway | 34.68111 | 0.027778 | 0.000187 | 0.024493 | LCK, PIK3CD, ZAP70 | |
| GO:0010818 | T cell chemotaxis | 65.89412 | 0.009259 | 0.000693 | 0.000693 | PIK3CD |  |
| GO:0035556 | intracellular signal transduction | 10.45938 | 0.008929 | 0.001473 | 0.001473 | LCK, LAT |  |
| GO:0003682 | chromatin binding | 11.16849 | 0.018519 | 0.001656 | 0.001656 | APBB1, SPI1, TAL1 | |
| GO:0043274 | phospholipase binding | 50.68778 | 0.008929 | 0.001713 | 0.001713 | LCK |  |
| GO:0000976 | transcription cis-regulatory region binding | 9.983957 | 0.009259 | 0.00174 | 0.00174 | SPI1, TAL1 | TCF7L2 |
| GO:0000785 | chromatin | 8.632417 | 0.018519 | 0.002156 | 0.002156 | SPI1, TAL1 | TCF7L2 |
| GO:0050900 | leukocyte migration | 41.18382 | 0.008929 | 0.002306 | 0.002306 | LCK |  |
| GO:0002250 | adaptive immune response | 52.71529 | 0.018519 | 0.002498 | 0.002498 | PIK3CD, ZAP70 | |
| GO:0014065 | phosphatidylinositol 3-kinase signaling | 25.34389 | 0.018519 | 0.002498 | 0.002498 | PIK3CD |  |
| GO:0030593 | neutrophil chemotaxis | 22.72211 | 0.009259 | 0.002498 | 0.002498 | PIK3CD |  |
| GO:0042826 | histone deacetylase binding | 15.68908 | 0.018519 | 0.002598 | 0.002598 | SPI1, TAL1 | |
| GO:0016310 | phosphorylation | 19.96791 | 0.009259 | 0.002707 | 0.002707 | PIK3CD |  |
| GO:0016301 | kinase activity | 33.79186 | 0.009259 | 0.003256 | 0.003256 | DGKA, PIK3CD | |
| GO:0001819 | positive regulation of cytokine production | 14.02003 | 0.009259 | 0.004155 | 0.004155 | PIK3CD |  |
| GO:0031295 | T cell costimulation | 24.40523 | 0.008929 | 0.005987 | 0.005987 | LCK |  |
| GO:0010595 | positive regulation of endothelial cell migration | 21.96471 | 0.027778 | 0.006046 | 0.006046 | PIK3CD, BCAR1 | |
| GO:0001772 | immunological synapse | 41.18382 | 0.009259 | 0.006918 | 0.035245 | LCK, LAT |  |
| GO:0030027 | lamellipodium | 11.26395 | 0.008929 | 0.00707 | 0.00707 | APBB1, RAC2 | |
| GO:0001784 | phosphotyrosine residue binding | 38.76125 | 0.046296 | 0.007171 | 0.011484 | LCK, ZAP70 | |
| GO:1902895 | positive regulation of miRNA transcription | 17.34056 | 0.009259 | 0.007201 | 0.007201 | SPI1 |  |
| GO:0030217 | T cell differentiation | 65.89412 | 0.009259 | 0.009994 | 0.009994 | LCK, PIK3CD | |
| GO:0042113 | B cell activation | 54.91176 | 0.009259 | 0.010394 | 0.010394 | PIK3CD, ZAP70 | |
| GO:0050853 | B cell receptor signaling pathway | 50.68778 | 0.009259 | 0.010394 | 0.010394 | PIK3CD, BCAR1 | |
| GO:0038083 | peptidyl-tyrosine autophosphorylation | 50.68778 | 0.009259 | 0.010976 | 0.010976 | LCK |  |
| GO:0051897 | positive regulation of protein kinase B signaling | 14.64314 | 0.009259 | 0.012301 | 0.012301 | PIK3CD | TCF7L2 |
| GO:0008180 | COP9 signalosome | 47.06723 | 0.008929 | 0.013991 | 0.013991 | LAT |  |
| GO:0045087 | innate immune response | 6.335973 | 0.009259 | 0.014948 | 0.014948 | PIK3CD |  |
| GO:0007173 | epidermal growth factor receptor signaling pathway | 18.82689 | 0.009259 | 0.015209 | 0.035245 | BCAR1 |  |
| GO:0006954 | inflammatory response | 5.883403 | 0.009259 | 0.015899 | 0.015899 | PIK3CD |  |
| GO:0008286 | insulin receptor signaling pathway | 19.38062 | 0.008929 | 0.018251 | 0.018251 | BCAR1 |  |
| GO:0090023 | positive regulation of neutrophil chemotaxis | 36.60784 | 0.009259 | 0.019429 | 0.019429 | RAC2 |  |
| GO:0010592 | positive regulation of lamellipodium assembly | 28.64962 | 0.009259 | 0.019429 | 0.019429 | RAC2 |  |
| GO:2001237 | negative regulation of extrinsic apoptotic signaling pathway | 25.34389 | 0.009259 | 0.019987 | 0.019987 |  | TCF7L2 |
| GO:0007265 | Ras protein signal transduction | 31.37815 | 0.009259 | 0.02199 | 0.040075 | KSR1, LAT |  |
| GO:0005667 | transcription regulator complex | 16.27015 | 0.009259 | 0.026706 | 0.026706 | SPI1, TAL1 | |
| GO:0042169 | SH2 domain binding | 21.96471 | 0.009259 | 0.026746 | 0.026746 | LCK |  |
| GO:0007229 | integrin-mediated signaling pathway | 23.9615 | 0.017857 | 0.027065 | 0.027065 | BCAR1, LAT | |
| GO:0019903 | protein phosphatase binding | 9.151961 | 0.009259 | 0.031353 | 0.031353 | LCK |  |
| GO:0007015 | actin filament organization | 36.60784 | 0.037037 | 0.032203 | 0.032203 | RAC2, BCAR1 | |
| GO:0000981 | DNA-binding transcription factor activity, RNA polymerase II-specific | 6.460208 | 0.009259 | 0.035039 | 0.035039 | SPI1 |  |
| GO:0060090 | molecular adaptor activity | 12.20261 | 0.009259 | 0.035361 | 0.035361 | SPI1 | TCF7L2 |
| GO:0030168 | platelet activation | 34.68111 | 0.018519 | 0.035734 | 0.043204 | DGKA, LCK | |
| GO:0005884 | actin filament | 14.02003 | 0.009259 | 0.041388 | 0.041388 | RAC2 |  |
| GO:0101003 | ficolin-1-rich granule membrane | 11.56037 | 0.009259 | 0.045108 | 0.045108 | AP2A2 |  |
| GO:0008013 | beta-catenin binding | 9.834943 | 0.009259 | 0.04797 | 0.04797 |  | TCF7L2 |
| GO:0051117 | ATPase binding | 9.549872 | 0.009259 | 0.04797 | 0.04797 | LCK |  |
| GO:0001558 | regulation of cell growth | 38.76125 | 0.008929 | 0.048304 | 0.048304 | BCAR1, TMC8 | |

**Table S11**. Reactome pathway enrichment analysis of the gene set shows which biological pathways are significantly enriched (over-represented) among the genes tested. Columns show the pathway identifier and description (Term_Description), Fold_Enrichment (relative over-representation compared with background), Support (proportion of genes from the dataset mapping to the pathway), lowest_p and highest_p (significance values), and the specific genes that were up- or down-regulated contributing to each pathway. **Reactome pathway enrichment** analysis revealed significant over-representation of immune-related signaling pathways, particularly those involving T-cell activation and co-stimulation (e.g., TCR signaling, CD28 pathways, CTLA4 inhibitory signaling), alongside PI3K/AKT signaling cascades. Central genes driving these enrichments included LCK, LAT, ZAP70, PIK3CD, and RAC2, which appeared repeatedly across multiple significant pathways

| ID | Term_Description | Fold_Enrichment | support | lowest_p | highest_p | Up_regulated | Down_regulated |
| --- | --- | --- | --- | --- | --- | --- | --- |
| R-HSA-114604 | GPVI-mediated activation cascade | 61.77574 | 0.074074 | 8.02E-08 | 3.31E-06 | LAT, LCK, RAC2 | |
| R-HSA-2219530 | Constitutive Signaling by Aberrant PI3K in Cancer | 25.34389 | 0.055556 | 2.12E-07 | 2.12E-07 | LCK, PIK3CD, RAC2 | |
| R-HSA-2219528 | PI3K/AKT Signaling in Cancer | 18.82689 | 0.046296 | 2.40E-07 | 2.40E-07 | LCK, PIK3CD, RAC2 | |
| R-HSA-6811558 | PI5P, PP2A and IER3 Regulate PI3K/AKT Signaling | 18.64928 | 0.055556 | 2.40E-07 | 2.40E-07 | LCK, PIK3CD, RAC2 | |
| R-HSA-199418 | Negative regulation of the PI3K/AKT network | 17.49401 | 0.055556 | 2.40E-07 | 2.40E-07 | LCK, PIK3CD, RAC2 | |
| R-HSA-1257604 | PIP3 activates AKT signaling | 6.936223 | 0.018519 | 6.30E-06 | 6.30E-06 | LCK, PIK3CD, RAC2 | |
| R-HSA-451927 | Interleukin-2 family signaling | 29.95187 | 0.009259 | 6.30E-06 | 6.30E-06 | LCK, PIK3CD | |
| R-HSA-9616222 | Transcriptional regulation of granulopoiesis | 42.51233 | 0.027778 | 1.50E-05 | 1.50E-05 | SPI1, TAL1 | |
| R-HSA-2424491 | DAP12 signaling | 45.44422 | 0.018519 | 3.04E-05 | 0.001082 | LAT, LCK |  |
| R-HSA-202433 | Generation of second messenger molecules | 53.42766 | 0.018519 | 4.31E-05 | 0.000353 | LAT, LCK, ZAP70 | |
| R-HSA-2172127 | DAP12 interactions | 31.37815 | 0.018519 | 4.77E-05 | 0.001856 | LAT, LCK |  |
| R-HSA-76002 | Platelet activation, signaling and aggregation | 12.97128 | 0.046296 | 7.43E-05 | 0.000123 | BCAR1, DGKA, LAT, LCK, RAC2 | |
| R-HSA-8939236 | RUNX1 regulates transcription of genes involved in differentiation of HSCs | 18.82689 | 0.037037 | 9.11E-05 | 9.11E-05 | SPI1, TAL1 | |
| R-HSA-202403 | TCR signaling | 16.33738 | 0.027778 | 0.000475 | 0.004266 | LAT, LCK, ZAP70 | |
| R-HSA-389359 | CD28 dependent Vav1 pathway | 54.91176 | 0.008929 | 0.000513 | 0.000513 | LCK |  |
| R-HSA-210990 | PECAM1 interactions | 54.91176 | 0.008929 | 0.000513 | 0.000513 | LCK |  |
| R-HSA-912631 | Regulation of signaling by CBL | 36.60784 | 0.018519 | 0.000709 | 0.000709 | PIK3CD |  |
| R-HSA-9669938 | Signaling by KIT in disease | 32.94706 | 0.009259 | 0.000709 | 0.000709 | LCK |  |
| R-HSA-9670439 | Signaling by phosphorylated juxtamembrane, extracellular and kinase domain KIT mutants | 32.94706 | 0.009259 | 0.000709 | 0.000709 | LCK |  |
| R-HSA-389357 | CD28 dependent PI3K/Akt signaling | 27.45588 | 0.009259 | 0.000858 | 0.000858 | LCK |  |
| R-HSA-1433559 | Regulation of KIT signaling | 41.18382 | 0.017857 | 0.000861 | 0.000861 | LCK |  |
| R-HSA-912526 | Interleukin receptor SHC signaling | 24.40523 | 0.009259 | 0.001007 | 0.001007 | PIK3CD |  |
| R-HSA-8878171 | Transcriptional regulation by RUNX1 | 7.162404 | 0.027778 | 0.001129 | 0.001129 | SPI1, TAL1 | |
| R-HSA-389513 | CTLA4 inhibitory signaling | 31.37815 | 0.008929 | 0.001223 | 0.001223 | LCK |  |
| R-HSA-9027276 | Erythropoietin activates Phosphoinositide-3-kinase (PI3K) | 54.91176 | 0.009259 | 0.001233 | 0.001233 | PIK3CD |  |
| R-HSA-202430 | Translocation of ZAP-70 to Immunological synapse | 59.90374 | 0.009259 | 0.001266 | 0.007155 | LCK, ZAP70 | |
| R-HSA-389356 | CD28 co-stimulation | 18.82689 | 0.009259 | 0.001479 | 0.001479 | LCK |  |
| R-HSA-167590 | Nef Mediated CD4 Down-regulation | 131.7882 | 0.037037 | 0.001621 | 0.001621 | AP2A2, LCK | |
| R-HSA-162909 | Host Interactions of HIV factors | 9.834943 | 0.018519 | 0.001621 | 0.001621 | AP2A2, LCK | |
| R-HSA-9706019 | RHOBTB3 ATPase cycle | 65.89412 | 0.009259 | 0.001681 | 0.001681 | PLIN3 |  |
| R-HSA-8853659 | RET signaling | 16.07174 | 0.009259 | 0.001856 | 0.001856 | PIK3CD |  |
| R-HSA-1433557 | Signaling by SCF-KIT | 15.32421 | 0.009259 | 0.001856 | 0.001856 | LCK |  |
| R-HSA-512988 | Interleukin-3, Interleukin-5 and GM-CSF signaling | 14.97594 | 0.018519 | 0.001856 | 0.001856 | PIK3CD |  |
| R-HSA-164952 | The role of Nef in HIV-1 replication and disease pathogenesis | 45.44422 | 0.046296 | 0.00199 | 0.004204 | AP2A2, LCK | |
| R-HSA-4086400 | PCP/CE pathway | 14.48222 | 0.037037 | 0.002009 | 0.002009 | AP2A2, RAC2 | |
| R-HSA-8866427 | VLDLR internalisation and degradation | 41.18382 | 0.009259 | 0.002488 | 0.002488 | AP2A2 |  |
| R-HSA-1660499 | Synthesis of PIPs at the plasma membrane | 12.43285 | 0.018519 | 0.002567 | 0.002567 | PIK3CD |  |
| R-HSA-9013407 | RHOH GTPase cycle | 36.60784 | 0.009259 | 0.002791 | 0.015275 | LCK, ZAP70 | |
| R-HSA-9006335 | Signaling by Erythropoietin | 26.35765 | 0.018519 | 0.002801 | 0.002801 | PIK3CD |  |
| R-HSA-3858494 | Beta-catenin independent WNT signaling | 13.63327 | 0.027778 | 0.00283 | 0.00283 | AP2A2, RAC2 | TCF7L2 |
| R-HSA-8849471 | PTK6 Regulates RHO GTPases, RAS GTPase and MAP kinases | 47.06723 | 0.018519 | 0.00283 | 0.00283 | BCAR1 |  |
| R-HSA-9607240 | FLT3 Signaling | 17.34056 | 0.009259 | 0.002843 | 0.005455 | LCK |  |
| R-HSA-171007 | p38MAPK events | 50.68778 | 0.018519 | 0.002913 | 0.002913 | RALGDS |  |
| R-HSA-372708 | p130Cas linkage to MAPK signaling for integrins | 43.92941 | 0.018519 | 0.003172 | 0.003172 | BCAR1 |  |
| R-HSA-164938 | Nef-mediates down modulation of cell surface receptors by recruiting them to clathrin adapters | 59.90374 | 0.037037 | 0.003418 | 0.003418 | AP2A2, LCK | |
| R-HSA-162906 | HIV Infection | 5.656147 | 0.009259 | 0.003418 | 0.003418 | AP2A2, LCK | |
| R-HSA-167044 | Signalling to RAS | 32.94706 | 0.018519 | 0.003546 | 0.003546 | RALGDS |  |
| R-HSA-5099900 | WNT5A-dependent internalization of FZD4 | 43.92941 | 0.009259 | 0.003921 | 0.003921 | AP2A2 |  |
| R-HSA-6802948 | Signaling by high-kinase activity BRAF mutants | 19.38062 | 0.009259 | 0.004208 | 0.004208 | KSR1 |  |
| R-HSA-5674135 | MAP2K and MAPK activation | 17.34056 | 0.009259 | 0.004208 | 0.004208 | KSR1 |  |
| R-HSA-9656223 | Signaling by RAF1 mutants | 16.89593 | 0.009259 | 0.004208 | 0.004208 | KSR1 |  |
| R-HSA-5675221 | Negative regulation of MAPK pathway | 15.32421 | 0.009259 | 0.004208 | 0.004208 | KSR1 |  |
| R-HSA-6802955 | Paradoxical activation of RAF signaling by kinase inactive BRAF | 15.32421 | 0.009259 | 0.004208 | 0.004208 | KSR1 |  |
| R-HSA-6802949 | Signaling by RAS mutants | 15.32421 | 0.009259 | 0.004208 | 0.004208 | KSR1 |  |
| R-HSA-6802946 | Signaling by moderate kinase activity BRAF mutants | 15.32421 | 0.009259 | 0.004208 | 0.004208 | KSR1 |  |
| R-HSA-9649948 | Signaling downstream of RAS mutants | 15.32421 | 0.009259 | 0.004208 | 0.004208 | KSR1 |  |
| R-HSA-354192 | Integrin signaling | 24.40523 | 0.018519 | 0.004366 | 0.005045 | BCAR1 |  |
| R-HSA-388841 | Costimulation by the CD28 family | 8.90461 | 0.009259 | 0.004786 | 0.004786 | LCK |  |
| R-HSA-186763 | Downstream signal transduction | 22.72211 | 0.009259 | 0.005045 | 0.005045 | BCAR1 |  |
| R-HSA-449836 | Other interleukin signaling | 27.45588 | 0.009259 | 0.005154 | 0.005154 | IL34 |  |
| R-HSA-187687 | Signalling to ERKs | 19.38062 | 0.027778 | 0.005231 | 0.005231 | RALGDS |  |
| R-HSA-8964043 | Plasma lipoprotein clearance | 17.80922 | 0.009259 | 0.005455 | 0.005455 | AP2A2 |  |
| R-HSA-1483255 | PI Metabolism | 7.844538 | 0.018519 | 0.005892 | 0.005892 | PIK3CD |  |
| R-HSA-9013404 | RAC2 GTPase cycle | 7.662107 | 0.027778 | 0.005909 | 0.005909 | RAC2 |  |
| R-HSA-76009 | Platelet Aggregation (Plug Formation) | 17.80922 | 0.018519 | 0.006209 | 0.006892 | BCAR1 |  |
| R-HSA-5668599 | RHO GTPases Activate NADPH Oxidases | 27.45588 | 0.009259 | 0.006429 | 0.006429 | RAC2 |  |
| R-HSA-202424 | Downstream TCR signaling | 6.655971 | 0.018519 | 0.00751 | 0.00751 | LCK |  |
| R-HSA-6802952 | Signaling by BRAF and RAF1 fusions | 10.62808 | 0.009259 | 0.007824 | 0.007824 | KSR1 |  |
| R-HSA-9613829 | Chaperone Mediated Autophagy | 31.37815 | 0.009259 | 0.007839 | 0.007839 | PLIN3 |  |
| R-HSA-186797 | Signaling by PDGF | 12.43285 | 0.009259 | 0.009205 | 0.009205 | BCAR1 |  |
| R-HSA-9006927 | Signaling by Non-Receptor Tyrosine Kinases | 11.98075 | 0.027778 | 0.009205 | 0.009205 | BCAR1 |  |
| R-HSA-8848021 | Signaling by PTK6 | 11.98075 | 0.027778 | 0.009205 | 0.009205 | BCAR1 |  |
| R-HSA-6802957 | Oncogenic MAPK signaling | 8.341028 | 0.009259 | 0.011461 | 0.011461 | KSR1 |  |
| R-HSA-3928665 | EPH-ephrin mediated repulsion of cells | 13.44778 | 0.009259 | 0.013656 | 0.013656 | AP2A2 |  |
| R-HSA-174824 | Plasma lipoprotein assembly, remodeling, and clearance | 8.90461 | 0.009259 | 0.016714 | 0.016714 | AP2A2 |  |
| R-HSA-202733 | Cell surface interactions at the vascular wall | 3.765378 | 0.009259 | 0.021658 | 0.021658 | LCK |  |
| R-HSA-6811440 | Retrograde transport at the Trans-Golgi-Network | 13.44778 | 0.027778 | 0.021911 | 0.021911 | PLIN3 |  |
| R-HSA-2871809 | FCERI mediated Ca+2 mobilization | 9.549872 | 0.009259 | 0.022379 | 0.02247 | LAT |  |
| R-HSA-2871796 | FCERI mediated MAPK activation | 9.413445 | 0.009259 | 0.022379 | 0.02247 | LAT |  |
| R-HSA-8856825 | Cargo recognition for clathrin-mediated endocytosis | 6.397487 | 0.009259 | 0.02494 | 0.02494 | AP2A2 |  |
| R-HSA-5673001 | RAF/MAP kinase cascade | 7.188449 | 0.009259 | 0.025076 | 0.025076 | KSR1, LAT, RALGDS | |
| R-HSA-5684996 | MAPK1/MAPK3 signaling | 7.034959 | 0.009259 | 0.025076 | 0.025076 | KSR1, LAT, RALGDS | |
| R-HSA-4420097 | VEGFA-VEGFR2 Pathway | 6.793208 | 0.018519 | 0.026139 | 0.026139 | BCAR1 |  |
| R-HSA-1483257 | Phospholipid metabolism | 6.589412 | 0.009259 | 0.027219 | 0.027219 | PIK3CD, PLA2G3 | |
| R-HSA-194138 | Signaling by VEGF | 6.27563 | 0.018519 | 0.028078 | 0.028078 | BCAR1 |  |
| R-HSA-2682334 | EPH-Ephrin signaling | 7.162404 | 0.009259 | 0.030205 | 0.030205 | AP2A2 |  |
| R-HSA-373760 | L1CAM interactions | 6.793208 | 0.009259 | 0.030205 | 0.030205 | AP2A2 |  |
| R-HSA-8856828 | Clathrin-mediated endocytosis | 4.673342 | 0.009259 | 0.040122 | 0.040122 | AP2A2 |  |
| R-HSA-166520 | Signaling by NTRKs | 9.834943 | 0.009259 | 0.040974 | 0.040974 | AP2A2, RALGDS | |
| R-HSA-6809371 | Formation of the cornified envelope | 9.413445 | 0.009259 | 0.045031 | 0.045031 | LCE5A |  |
| R-HSA-187037 | Signaling by NTRK1 (TRKA) | 11.45985 | 0.009259 | 0.048662 | 0.048662 | AP2A2, RALGDS | |
| R-HSA-2454202 | Fc epsilon receptor (FCERI) signaling | 3.853457 | 0.008929 | 0.049289 | 0.049289 | LAT |  |
| T**erm_Description** – the Reactome pathway name. **Fold_Enrichment** – how much more represented this pathway is compared to chance. Higher = stronger over-representation. **Support** – the proportion or count of genes from your dataset that map to that pathway. **lowest_p / highest_p** – statistical significance (adjusted p-values). Smaller = more robust enrichment. **Up_regulated / Down_regulated** – genes from your dataset that drive the enrichment. | | | | | | | |

References

1.	Trichopoulou A, Costacou T, Bamia C, Trichopoulos D. Adherence to a Mediterranean diet and survival in a Greek population. N Engl J Med. 2003;348(26):2599-608.

2.	Black LJ, Baker K, Ponsonby AL, van der Mei I, Lucas RM, Pereira G. A Higher Mediterranean Diet Score, Including Unprocessed Red Meat, Is Associated with Reduced Risk of Central Nervous System Demyelination in a Case-Control Study of Australian Adults. The Journal of nutrition. 2019;149(8):1385-92.
